# Scenario-based portfolio optimization via bootstrapping and machine learning methods: Theory development and empirical evidence from the Tehran Stock Market

**DOI:** 10.1371/journal.pone.0342593

**Published:** 2026-02-19

**Authors:** Morteza Amini, Sajedeh Javadi, Majid Soleimani-damaneh

**Affiliations:** School of Mathematics, Statistics and Computer Science, College of Science, University of Tehran, Tehran, Iran; Memorial Sloan Kettering Cancer Center, UNITED STATES OF AMERICA

## Abstract

Predicting future returns and modeling return uncertainty are essential yet challenging tasks in portfolio optimization. To address these issues, this study proposes a hybrid approach combining machine learning for return prediction, bootstrapping for uncertainty modeling, and scenario optimization for portfolio selection in the presence of uncertainty. Bootstrap samples are used to generate multiple return trajectories via machine learning, with each trajectory treated as a distinct scenario in a scenario-based mean-variance optimization framework. Empirical results using data from the Tehran Stock Market demonstrate that the proposed model produces optimal portfolios that align more closely with those derived from actual prices, compared to those generated by traditional techniques. Our results show that the combination of bootstrapping, machine learning-based prediction, and scenario optimization is a compelling alternative to classical methods. Indeed, by combining bootstrapping, as a non-parametric method for uncertainty quantification, with machine learning, the model avoids strong distributional assumptions (e.g., normality of returns) and works without assuming a predefined form of uncertainty set. In addition to illustrating the advantages of our approach by implementing it on real-world datasets, we theoretically prove that the resulting scenario optimization problem is a convex program that generates efficient solutions superior to those produced by worst-case robust optimization techniques. Furthermore, the developed framework can accommodate different machine learning models and bootstrapping techniques. Moreover, the use of scenario optimization is computationally tractable and aligns with even large-scale projects.

## 1 Introduction

Portfolio optimization aims to maximize the expected return while minimizing financial risk, offering a systematic framework for wealth allocation that seeks an optimal balance between return and risk. Markowitz [1] laid the foundation for modern portfolio theory by introducing the Mean-Variance (MV) model. The MV model constructs an optimal portfolio by maximizing expected return for a given level of risk, or minimizing risk for a target expected return, where the variance of portfolio returns measures risk. There have been several alternatives in the literature for MV model, including, among others, the Capital Asset Pricing Model [2], the Mean Absolute Deviation [3], VAR-based model [4], probabilistic risk measurement model [5], and the $\ell_{\infty }$ model [6]. Despite the aforementioned advancements, the MV model continues to serve as a cornerstone in financial research and practice due to its simplicity, tractability, and foundational role in modern portfolio theory (see e.g., [7–12]).

The classical portfolio optimization framework obtains the optimal portfolio based on historical data (see, e.g., [7,9,11,13]), while in most applications, investors are primarily interested in using these models to make future investment decisions. To handle this issue, forecasting future asset returns and the construction of the optimal portfolio based on the predicted returns has emerged as a viable and effective approach. A variety of machine learning models are used by scholars to predict future asset prices along with a portfolio optimization method to construct the optimal portfolio. A detailed literature review of these methods is presented in Sect 2.

A serious challenge in the construction of an optimal portfolio based on the predicted return is the high level of uncertainty caused by the prediction. This uncertainty along with the uncertainty caused by estimation of the parameters (means and covariances for the MV model), significantly decreases the reliability of the derived portfolio. So, a substantial body of research has investigated the impact of parameter uncertainty on portfolio optimization. Barry [14] analyzed the robustness of optimal portfolios under the MV framework and found that, although the efficient set may remain relatively stable, the associated risks can vary considerably. Michaud [13] demonstrated that portfolios derived from the MV model are highly unstable, and small changes in input estimates can drastically alter portfolio composition. Fabozzi et al. [15] examined the inherent vulnerability of classical portfolio optimization frameworks to parameter estimation errors and model uncertainty, emphasizing the need for more robust approaches in practical applications. Bodnar and Schmid [7] examined the implications of parameter uncertainty by deriving the empirical distributions of portfolio returns and risks based on sample estimates. Their findings revealed significant discrepancies between these empirical distributions and the assumptions underlying the classical model. Lai et al. [9] argued that estimation errors in the mean and covariance matrix result in poor performance and substantial optimization error under the MV model. Tu and Zhou [11] further showed that simple allocation strategies can, in some cases, outperform more sophisticated optimization-based approaches that rely heavily on historical data, primarily due to their lower sensitivity to parameter estimation errors.

To handle uncertainty two major approaches have been developed: Robust Optimization (RO) and Scenario Optimization (SO), both aim to explicitly incorporate uncertainty into the portfolio selection process and to enhance the stability and robustness of the resulting portfolios. Ben-Tal and Nemirovski [16] introduced a foundational framework for RO, addressing the sensitivity of Linear Programming (LP) solutions to even minor uncertainties in input data. Goldfarb and Iyengar [17] advanced the theory of robust portfolio optimization by addressing the sensitivity of the classical MV model to estimation errors in key parameters such as expected return and covariance matrix. They proposed a factor-based RO framework in which the uncertainty in model parameters is captured using statistically grounded confidence sets. Fabozzi et al. [15] introduced a robust MV portfolio optimization model that explicitly accounts for parameter uncertainty by defining uncertainty sets, thereby improving the model’s stability and performance under estimation errors. Pınar [18] extended the classical MV model by incorporating ellipsoidal uncertainty in the expected returns and derived closed-form solutions for robust portfolio optimization. Ismail and Pham [8] introduced a robust Markowitz MV portfolio selection approach under an ambiguous covariance matrix. Rahimi and Soleimani-damaneh [19] modeled uncertainty in portfolio selection via dual ordering cones in vector optimization. Koushki et al. [20] developed an interactive algorithm to support a decision maker in finding a most preferred lightly robust efficient portfolio.

While RO provides powerful tools for guaranteeing solution feasibility and performance under parameter uncertainty, this method focuses on the absolute worst case, which sometimes might lead to overly conservative decisions that may significantly compromise performance under more typical or likely conditions [21,22]. Furthermore, it has been revealed that focusing solely on the worst-case scenario might lead to non-optimal decisions if a non-worst-case scenario materializes [22,23]. Moreover, precisely defining the complete uncertainty set required by robust methods is challenging in practice.

Another promising tool for handling uncertainty is SO, first introduced by Dembo [24]. This approach integrates solutions from individual scenarios into a single, implementable policy. The SO approach involves a two-stage process. In the first stage, deterministic optimization problems are solved independently for each defined scenario, where each scenario represents a specific realization of the uncertain parameters. This stage yields a set of individual scenario optimal solutions and their corresponding objectives. In the second stage, a tracking model is formulated and solved, which aims to synthesize the information from the individual scenario solutions into a single, implementable decision policy. SO offers several advantages over classical probabilistic approaches. It does not require precise knowledge of the underlying probability distributions for uncertain parameters and, unlike traditional stochastic methods, can be readily applied to nonlinear models. Furthermore, this approach is highly versatile and can accommodate different sources of uncertainty, in expected returns, covariances, asset prices, and other parameters. Since scenarios are often generated from historical data or empirical simulations, they tend to reflect actual market conditions more accurately, thereby enhancing the practical relevance of the resulting portfolio decisions. In recent years, numerous scholars have applied SO approaches across various domains, including financial problems. For example, Mausser and Rosen [25] employed an SO framework for credit risk portfolio optimization rather than relying on probability distribution (which may be poorly specified). Barro and Canestrelli [26] employed scenarios as inputs to a multi-stage stochastic programming problem. They represented future uncertainty (including asset prices and returns) through a finite set of scenarios, and then a Progressive Hedging Algorithm was applied to decompose the original problem into smaller subproblems, each corresponding to a specific scenario. Pagnoncelli et al. [27] applied SO to chance-constrained programming to achieve a better balance between risk and return.

Another tool that helps in handling uncertainty is bootstrapping [28], a statistical resampling technique introduced by Efron [29]. Resampling with replacement from the observed data generates multiple bootstrap samples to approximate the uncertainty measures. This data-driven approach provides a powerful and versatile tool for quantifying the uncertainty inherent in statistical estimates without relying on strong distributional assumptions. The reader is referred to [30] for more details and the theory of bootstrapping.

In this study, we propose the use of bootstrapping as a resampling statistical method to model uncertainty in parameters of the MV model and incorporate scenario optimization to handle this uncertainty by averaging over different scenarios provided by the bootstrap samples. Two well-known and common machine learning methods, Convolutional Neural Networks (CNN) and Gated Recurrent Units (GRU) are used to predict the future returns, based on historical data. Our empirical results demonstrate that the portfolio constructed using the SO approach aligns more closely with the portfolio derived from true prices, compared to the portfolio generated by the MV model without bootstrapping. This finding highlights the effectiveness of incorporating prediction uncertainty into the portfolio construction process through a scenario-based framework. Our analysis demonstrates that integrating bootstrapping with machine learning-based prediction and SO offers a robust alternative to traditional portfolio optimization approaches. This combined methodology leverages bootstrapping as a non-parametric technique for uncertainty quantification while eliminating restrictive distributional assumptions (such as requiring normal return distributions) and avoiding predefined uncertainty set formulations. The proposed framework provides flexibility to incorporate various machine learning algorithms and bootstrapping variants. Additionally, the scenario optimization component maintains computational efficiency, making it suitable for implementation in large-scale applications. In addition to demonstrating the benefits of our approach through experiments on real-world datasets, we provide a theoretical proof that the resulting scenario optimization problem is convex and yields efficient solutions that outperform those obtained via worst-case robust optimization methods.

The remainder of the paper is organized as follows. Sect 2 reviews price prediction using machine learning methods. sec3Sect 3 introduces the key methods, concepts, and datasets used in our study, along with their preprocessing steps. The proposed bootstrap-based prediction methods are presented in Sect 4. Sect 5 proposes the scenario-based formulation of the MV model and reports empirical results from applying the models to the data, including a detailed analysis. In Sect 6, we present theoretical results demonstrating the superiority of the proposed approach compared with the worst-case robust optimization method. Finally, Sect 7 concludes the paper by summarizing key findings and suggesting directions for future research. Before proceeding with this paper, readers are encouraged to review the comprehensive list of abbreviations provided in Table 1 to facilitate reading comprehension.

**Table 1:** Glossary of abbreviations (alphabetically ordered).

Abbreviation	Full Term
ANN	Artificial Neural Network
BTC	Bitcoin
CNN	Convolutional Neural Networks
DT	Decision Tree
ETH	Ethereum
GA	Genetic Algorithm
GRU	Gated Recurrent Units
kNN	k-Nearest Neighbors
LP	Linear Programming
LR	Logistic Regression
LSTM	Long Short-Term Memory
LTC	Litecoin
MAE	Mean Absolute Error
MAPE	Mean Absolute Percentage Error
MSE	Mean Square Error
MV	Mean-Variance
PD	Portfolio Deviation
RF	Random Forest
RMSE	Root Mean Square Error
RNN	Recurrent Neural Network
RO	Robust Optimization
SO	Scenario Optimization
SGD	Stochastic Gradient Descent
SVM	Support Vector Machine
S-RNN	Simple RNN
SVR	Support Vector Regression

## 2 Review of price prediction using machine learning

The prediction of asset returns is consistently of interest to investors, as asset prices are influenced by many factors, including political conditions, corporate strategies and announcements, interest rate fluctuations, economic conditions, and investor sentiment [31]. A considerable body of research has utilized diverse machine learning models to predict asset returns. In this section, we review a selection of notable studies in this field.

Tay and Cao [32] applied Support Vector Machines (SVMs) to predict financial time series, applied to five real data sets collected from the Chicago Mercantile Market. Their findings revealed that SVM outperformed the Backpropagation neural network. Huang [33] proposed a hybrid asset selection model, GA-SVR, which integrates Genetic Algorithms (GA) with Support Vector Regression (SVR) for stock return prediction. In this approach, stocks are ranked based on the predicted returns, with higher-ranked stocks being selected for portfolio construction. Additionally, GA is employed for feature selection and the optimization of model parameters. Okasha [34] assessed the performance of the SVM in time series forecasting by comparing it with the ARIMA and Artificial Neural Network (ANN) models. Selven et al. [35] conducted a comparative analysis of Long Short-Term Memory (LSTM), Recurrent Neural Networks (RNN), and CNN models for predicting the stock prices of companies listed on the National Stock Exchange of India. Singh [36] examined the effectiveness of several popular machine learning algorithms, including AdaBoost, *k*-Nearest Neighbors (kNN), Logistic Regression (LR), ANN, Random Forest (RF), Stochastic Gradient Descent (SGD), SVM, and Decision Tree (DT), in predicting the closing price of the Nifty 50 stock index.

Kim and Kim [37] proposed a feature fusion LSTM-CNN model to enhance stock price prediction accuracy. Erbetsev et al. [38] investigated the use of ensemble learning algorithms [39], specifically RF and SGBM, for the prediction of the daily prices of cryptocurrencies. The results showed that both methods effectively captured the time series behavior of cryptocurrency prices. Moreover, in terms of accuracy metrics, namely Mean Absolute Percentage Error (MAPE) and Root Mean Square Error (RMSE), the ensemble algorithms outperformed baseline models such as linear regression and decision trees, indicating their superior predictive performance. Hmayel and Owad [40] demonstrated that LSTM, GRU, Bi-LSTM, and a hybrid model combining GRU and LSTM generally achieve lower RMSE and MAPE values in forecasting the prices of three major cryptocurrencies such as Litecoin (LTC), Ethereum (ETH), and Bitcoin (BTC), compared to traditional methods. Yoo and Lee [41] proposed a novel approach to portfolio management using threshold-based models, which are integrated with RNNs for stock range prediction. Their study examined three types of recurrent networks: simple RNN (S-RNN), LSTM, and GRU. The dataset consists of historical data from 10 stocks listed on the S&P-500 index. Mishra and Padhy [42] introduced a portfolio construction method based on stock price prediction using the SVR model. The study utilized weekly stock prices of 92 stocks from the BSE-30 and Nifty-100 indices. The optimal portfolio is then calculated using the MV model.

Wang et al. [43] proposed a hybrid model based on LSTM networks for the prediction of future stock returns and the MV model for portfolio optimization, to improve asset selection and capital allocation optimization. Their study utilized daily stock data from 21 assets listed in the FTSE 100 index. They compared the LSTM model with SVM, RF, DNN, and ARIMA. Ma et al. [44] proposed a novel approach to portfolio optimization by first predicting the daily returns of Chinese Stock Market assets using a range of machine learning algorithms. The predicted returns were then incorporated into a traditional optimization framework to determine the optimal portfolio weights. The results demonstrated that CNN and DNN models outperformed other methods in terms of prediction accuracy. Moreover, the portfolios constructed based on the predicted data yielded higher returns and risk performance compared to those derived from classical approaches. Mazzouz and Elmoufidi [45] conducted a comparative analysis of three models: GRU, LSTM, and 1-dimensional CNN (1D-CNN) for the prediction of the FTSE CSE index returns. Their results demonstrated that the GRU model consistently outperformed the other two in terms of predictive accuracy, providing more realistic predictions as Mean Squared Error (MSE) and RMSE. Table 2 presents a summary of the literature on stock return prediction using machine learning.

**Table 2:** Overview of studies on stock return prediction via machine learning.

Reference	Prediction Method	Data set	Period
[32]	SVM, PBNN	Five real futures contracts from the Chicago Mercantile Market	Dec 1992 - Jul 1996, Jan 1993 - Aug 1996, Jun 1995 - Feb 1999
[33]	SVR with GAs	The constituent stocks of the 200 largest market capitalization listed in the Taiwan Stock Exchange	1996 - 2010
[44]	RF, SVR, LSTM, DMLP, CNN	China securities 100 index	Jan 4, 2007 - Dec 31, 2015
[42]	SVR	BSE-30 & Nifty-100 (Indian stock market)	Jan 2010 - Dec 2015
[38]	RF, SGBM	Cryptocurrencies (BTC, Ripple (XRP), ETH)	Jan 1, 2015 - Dec 31, 2019 (for BTC and XRP), Aug 7, 2015 - Dec 31, 2019 (for ETH)
[41]	RNN, LSTM, GRU	The top 10 stocks in S&P 500 index	Jan 1997 - Dec 2016
[35]	LSTM, RNN, CNN	Three companies (Infosys, TCS, CIPLA) from NSE	Jul 2014 - Nove 2014
[43]	LSTM, SVM, DNN, RF, ARIMA	Twenty-one stocks from FTSE 100 (The UK Stock Exchange 100 Index)	1994 - Mar 2019
[36]	AdaBoost, kNN, LR, ANN, RF, SGD, SVM, DT	The Nifty 50 Index (Indian stock market)	Apr 22, 1996 - Apr 16, 2021
[37]	ST-LSTM, SC-CNN, LSTM-CNN	SPDR S&P 500 ETF Trust	Oct 14, 2016 - Oct 16, 2017
[40]	GRU, LSTM, bi-LSTM	Cryptocurrency (BTC, litecoin, ETH)	Jan 1, 2018- Jun 30, 2021
[45]	GRU, LSTM, 1D-CNN	The FTSE CSE index	-

As demonstrated in the related works reviewed above, deep learning techniques have shown significant advantages in forecasting asset returns, outperforming both traditional statistical models and classical machine learning approaches. Architectures such as LSTM, GRU, and CNN have been extensively applied to financial time series prediction and are consistently ranked among the most effective models in this domain [35,40,41,43,45]. Accordingly, in this study, we adopt CNN and GRU for return prediction, given their proven effectiveness. GRU is particularly chosen for its computational efficiency and simpler architecture compared to LSTM, while retaining comparable predictive capability.

## 3 Materials and methods

sec3 This section introduces the key methods and concepts used in our study. Additionally, we introduce the datasets employed and describe the preprocessing steps for preparing the data for analysis.

### 3.1 Scenario optimization

One of the earliest studies that introduced the concept of “ scenario optimization” was conducted by Dembo [24], who presented a method for solving optimization problems under uncertainty conditions. As a relevant example, consider the following linear problem:

$$\min_{x}\;c_{u}^{T}x\;s.t.\;A_{u}x=b_{u},\;A_{d}x=b_{d},\;B_{d}x\leq e_{d},$$ (1)

where *x* is the vector of decision variables, $A_{d}\in {\mathbb{R}}^{m_{1}\times N}$, $b_{d}\in {\mathbb{R}}^{m_{1}\times 1}$, $B_{d}\in {\mathbb{R}}^{m_{2}\times N}$, and $e_{d}\in {\mathbb{R}}^{m_{2}\times 1}$ are deterministic matrices, while $c_{u}\in {\mathbb{R}}^{N\times 1}$, $A_{u}\in {\mathbb{R}}^{m_{0}\times N}$, and $b_{u}\in {\mathbb{R}}^{m_{0}\times 1}$ are uncertain parameters. It is assumed that the uncertain parameters belong to a finite set of states, and each state of the parameters is considered as a scenario. The set of all scenarios is denoted by *S*. For each s∈S, problem (1) leads to a deterministic problem, referred to as the scenario subproblem

$$\hat{\nu_{s}}=\min_{x}\;\;\;c_{s}^{T}x\;\;\;\;s.t.\;\;\;\;A_{s}x=b_{s},\;\;\;\;\;A_{d}x=b_{d},\;\;\;\;\;B_{d}x\leq e_{d}.$$ (2)

The probability of scenario *s* is denoted *P*_*s*_ and $\sum_{s\in S}P_{s}=1$. The collection of all scenario subproblems corresponding to s∈S is referred to as the SO problem.

The most important question in SO is “ Just how should the solutions under several different scenarios be combined to form a single reasonable solution to the underlying stochastic problem?" [24]. There are various approaches to answering this question, leading to the development of a model for coordinating the solutions to problem (2), referred to as the coordinating model or tracking model. This SO approach consists of two steps:

**Step 1:** Compute solutions to the (deterministic) problem (2) for each scenario s∈S.

**Step 2:** Solve a tracking model to find a single feasible policy.

The critical feature of this approach is found in Step 2, where its exact formulation depends on the specific context under consideration. Nevertheless, the problems in Steps 1 and 2 are deterministic and can typically be solved using well-established methods. Dembo [24] defines stochastic feasibility as follows: a solution *x* to the stochastic linear program (1) is considered feasible if it solves the following problem.


$$\min_{x}\;\;\;\sum_{s\in S}P_{s}\|A_{s}x-b_{s}{\|}^{2}\;s.t.\;\;\;A_{d}x=b_{d},\;\;\;\;B_{d}x\leq e_{d}.$$

Furthermore, Dembo [24] introduced the following tracking model to integrate the solutions of problem (2) for all s∈S, thereby producing a unified solution:

$$\min_{x}\;\sum_{s\in S}P_{s}(\|c_{s}^{T}x-{\hat{v}}_{s}{\|}^{2}+\|A_{s}x-b_{s}{\|}^{2})\;\;s.t.\;\;\;\;\;\;\;A_{d}x=b_{d},\;\;\;\;\;\;B_{d}x\leq e_{d}.$$ (3)

By selecting different norms in (3), various tracking models can be formulated. Consequently, the tracking model does not have a unique formulation; however, it must adhere to the concept of stochastic feasibility as defined by Dembo [24].

### 3.2 Portfolio optimization using MV model

In financial markets, investors are typically faced with a wide range of risky investment assets. Given *N* such assets, the investor seeks to determine how to allocate initial capital (This denotes the available wealth designated by the investor for allocation across a portfolio of assets.) among them in a way that maximizes expected return while minimizing the investment risk.

Markowitz [1] conducted one of the earliest studies in the field of mathematical programming for portfolio selection by introducing the MV model, which aims to minimize risk for a given level of expected return (or maximize expected return for a specified level of risk). This model assumes that *x*_*j*_ denotes the proportion of initial capital allocated to the *j*th asset. Let *R*_*j*_ denote the random variable representing the return of asset *j*. The expected return of asset *j*, denoted by $\mu_{j}$, is the mathematical expectation of *R*_*j*_ ($\mu_{j}=E(R_{j})$). Therefore, the expected return of the portfolio $X=(x_{1},x_{2},...,x_{N})$ can be expressed as follows:


$$\text{Return}(x_{1},x_{2},\cdots ,x_{N})=E(\sum_{j=1}^{N}R_{j}x_{j})=\sum_{j=1}^{N}E\left(R_{j}\right)x_{j}=\sum_{j=1}^{N}\mu_{j}x_{j}.$$

Markowitz employed the variance of asset returns as a measure of risk. Consequently, the risk in the MV model can be defined as follows:


$$\text{Risk}(x_{1},x_{2},\cdots ,x_{N})=var(\sum_{j=1}^{N}R_{j}x_{j})=\sum_{j=1}^{N}\sigma_{j}^{2}x_{j}^{2}+2\sum_{1\leq i<j\leq N}\sigma_{ij}x_{i}x_{j}\;=\sum_{i=1}^{N}\sum_{j=1}^{N}\sigma_{ij}x_{i}x_{j},$$

where $\sigma_{j}^{2}$ is the variance of the random variable *R*_*j*_ and $\sigma_{ij}$ is the covariance between the two random variables *R*_*i*_ and *R*_*j*_. The MV model can be expressed as follows [12]:

$$\min\;\sum_{i=1}^{N}\sum_{j=1}^{N}\sigma_{ij}x_{i}x_{j},\;\;s.t.\;\;\sum_{j=1}^{N}\mu_{j}x_{j}\geq \rho ,\;\;\;\;\;\sum_{j=1}^{N}x_{j}=1,\;\;\;\;\;\;x_{j}\geq 0,\;j=1,2,\cdots ,N,$$ (4)

where *ρ* is the minimum expected return desired by the investor. Here, $x_{j}\geq 0$ indicates that short-selling (The portfolio weights must be non-negative, and investors are restricted to holding only long positions in the assets.) is not allowed.

The single-objective formulation presented in Problem (4), which minimizes portfolio risk subject to a specified level of expected return, does not follow the actual goals of investors. In practice, investors typically seek a trade-off between risk and return, maximizing expected return while simultaneously minimizing portfolio risk. Moreover, the traditional Markowitz approach to constructing the Pareto frontier requires considering multiple states [1], which becomes computationally impractical as the number of assets increases. To address this issue, the Markowitz portfolio problem can be reformulated within a multi-objective optimization framework (see e.g., [13,19,20,46,47]). Specifically, Problem (4) can be expressed as the following bi-objective model:

$$\min\;\sum_{i=1}^{N}\sum_{j=1}^{N}\sigma_{ij}x_{i}x_{j},\;\max\;\sum_{j=1}^{N}\mu_{j}x_{j},\;\;\;s.t.\;\;\;\;\;\;\;\sum_{j=1}^{N}x_{j}=1,\;\;\;x_{j}\geq 0,\;j=1,2,\cdots ,N$$ (5)

Using the weighted sum scalarization method in multi-objective optimization [48], Problem (5) is transformed to the following parametric problem:

$$\min\;\lambda \sum_{i=1}^{N}\sum_{j=1}^{N}\sigma_{ij}x_{i}x_{j}-(1-\lambda )\sum_{j=1}^{N}\mu_{j}x_{j},\;\;\;s.t.\;\;\;\;\;\;\;\sum_{j=1}^{N}x_{j}=1,\;\;\;x_{j}\geq 0,\;j=1,2,\cdots ,N$$ (6)

where *λ* is the risk aversion coefficient with λ∈[0,1]. Values of *λ* near 0, indicate that the investor is not risk-averse and pursues the maximization of return rather than considering risk. In contrast, values of *λ* near 1, mean that the investor seeks to minimize risk rather than considering return.

A matrix-vector formulation of Problem (6) can be expressed as

$$\min_{x}\;\lambda x^{T}\Sigma x-(1-\lambda )\mu^{T}x\;\text{s.t.}\;\;\;\;e^{T}x=1,\;\;\;\;x\geq 0,$$ (7)

where $\Sigma =[\sigma_{ij}{]}_{N\times N}$ denotes the variance-covariance matrix of asset returns, and $\mu =[\mu_{j}{]}_{N\times 1}$ represents the vector of expected returns. Furthermore, $e\in {\mathbb{R}}^{N}$ is a vector of ones, e.g., $e^{T}x=\sum_{j=1}^{N}x_{j}.$

### 3.3 Machine learning methods

As demonstrated by the related works reviewed in Sect 2, deep learning techniques have shown significant advantages in forecasting asset returns, outperforming both traditional statistical models and classical machine learning approaches. Architectures such as CNN and GRU have been extensively applied to financial time-series prediction and are consistently ranked among the most effective models in this domain [35,40,41,43,45]. Accordingly, this study adopts CNN and GRU for return prediction, given their proven effectiveness. GRU, described in the following, is particularly chosen for its computational efficiency and simple architecture while maintaining comparable predictive performance.

Our choice of 1D-CNN and GRU was a deliberate and strategic one, focused on establishing a robust and interpretable benchmark rather than providing an exhaustive comparison of all modern architectures. The 1D-CNN is exceptionally adept at learning local, multi-scale temporal patterns and salient features within time series, which are often critical in financial data. The GRU was selected as our primary recurrent unit because it captures long-term dependencies with computational efficiency comparable to more complex models. It is well established in the literature that GRUs, as a streamlined variant of the LSTM, often achieve comparable or superior performance due to their simpler gating mechanism, which reduces the risk of overfitting on finite financial datasets. Thus, employing a GRU inherently covers the capabilities of an LSTM while being more optimized for our context.

With respect to additional benchmarks, we emphasize that the aim of this study was not to perform an exhaustive comparative evaluation of all competing algorithms, but rather to rigorously assess the effectiveness of a targeted hybrid framework grounded in established deep-learning methodologies. Although Transformer-based architectures have demonstrated strong performance in a variety of domains, their application to financial time-series forecasting remains relatively limited and typically demands substantially large datasets to achieve competitive accuracy, which reduces their suitability as definitive baselines for our setting. Likewise, while XGBoost is a well-established and powerful method, its performance is highly sensitive to extensive hyperparameter tuning and relies on a fundamentally different, non-sequential inductive bias that is not directly comparable to the sequence-modeling paradigm employed in this work.

Our study establishes a solid foundation by demonstrating the effectiveness of a CNN or GRU architecture, a contribution that also facilitates future comparisons with more advanced models. It is important to emphasize, however, that the primary contribution of this work does not lie in the specific choice of machine-learning architecture, but in the novel integration of bootstrapping and scenario optimization to quantify uncertainty and enable risk-aware decision-making. Within this framework, the machine-learning model serves as a flexible forecasting component; therefore, its specific form is of secondary importance.

#### Convolutional Neural Networks (CNNs).

CNN, introduced by Lecun et al. [49], is widely used in image processing. These models can also be applied to univariate time series data using 1-dimensional kernels. CNNs have various types of layers, including convolutional layers, pooling layers, and fully connected layers. Convolutional layers use kernels to extract local features of the input. Pooling layers reduce the dimension of the outputs of the convolutional layers, to decrease model complexity and increase stability. The fully connected layers are used for the prediction task. More details about CNN, along with graphical illustrations of this deep learning technique, are provided in A.1Appendix A.

#### Gated Recurrent Unit (GRU).

GRU was introduced by Cho et al. [50] as an optimized alternative to the LSTM model. GRU is designed to model sequential data by allowing information to be selectively used or forgotten over time. The GRU has a simpler architecture than the LSTM, with fewer parameters, making it generally easier to train and more computationally efficient [51]. This network consists of cells with two gates: the reset gate and the update gate. The reset gate determines how much of the previous hidden state to forget, while the update gate determines how much of the candidate activation vectors to incorporate into the new hidden state. GRU has gained popularity due to its simplicity, efficiency, and effectiveness in various sequence-based tasks. More details about GRU, along with graphical illustrations of this technique, are provided in A.2Appendix B.

### 3.4 Tehran stock market data set

The proposed method is evaluated using two case studies. The first case study involves a portfolio of 9 assets over a 60-day horizon (with 883 price observations for each asset), and the second involves a portfolio of 15 assets over a 90-day horizon (with 1,678 price observations for each asset). All assets were chosen from the Tehran Stock Market website [52].

#### Case Study I.

The data used in this study includes the daily returns of nine assets from the Tehran Stock Market, selected based on the available continuous closing prices within a specified period. The assets included are Akhaber (Iran Telecommunication), Asia (Asia Insurance), Foulad (Mobarake Isfahan Steel), Hkshti (IRISL Group), Mafakher (Kharazmi Information Technology Development), Nori (Nori Petrochemical), Satran (Tehran Housing Investment), Shasta (Social Security Investment), and Tejarat (Tejarat Bank). The information for these selected assets was obtained from the Tehran Stock Market website [52]. The dataset includes the closing prices of these assets in working days from February 24, 2021, to December 31, 2023, amounting to 883 observations for each asset. The return of asset *j* on day *t* is computed as

$$R_{jt}=\frac{P_{j(t+1)}-P_{jt}}{P_{jt}},\;t=1,2,\ldots ,T=882,$$ (8)

where *P*_*jt*_ denotes the closing price of asset *j* on day *t*, and the expected return of the *j*th asset is estimated by


$$\mu_{j}=\frac{1}{T}\sum_{t=1}^{T}R_{jt}.$$

Furthermore, the covariance between the returns of assets *i* and *j* is estimated by


$$\sigma_{ij}=\frac{1}{T-1}\sum_{t=1}^{T}(R_{jt}-\mu_{j})(R_{it}-\mu_{i}).$$

To ensure the integrity of time series data and enhance the robustness of predictive modeling, anomalous price jumps, typically arising from events such as dividend distributions, capital increases, or technical corrections, were systematically filtered. Such events introduce artificial discontinuities in asset price movements, which can adversely affect model training and forecasting accuracy. Based on the trading rules of Tehran Stock Market, daily returns exceeding an absolute threshold of 10% were considered indicative of abnormal price jumps and were therefore excluded from the dataset.

To evaluate the performance of the proposed methods, we have separated the last 60 data points for each asset, which will be used for comparison with the algorithm outputs. Thus, we consider 823 data points for training the CNN and GRU models.

#### Case Study II.

The dataset for this case study comprises daily closing prices of 15 prominent companies listed on the Tehran Stock Market, covering a wide range of sectors, including automotive, steel, oil, cement, pharmaceuticals, and dairy. The selected companies are: Khodro (Iran Khodro Company), Foulad (Mobarake Isfahan Steel), Shepna (Isfahan Oil Refinery), Kgl (Golgohar Mining and Industrial Company), Sefars (Fars and Khuzestan Cement), Drouz (Rouz Darou Pharmaceutical), Bswitch (Pars Switch), Kkhak (Iran China Clay), Hkshti (IRISL Group), Ghshasfa (Pegah Isfahan Pasteurized Milk), Sakht (International Building Development), Ghsabet (Sabet Khorasan Sugar), Sesoufi (Soufian Cement), Khsapa (SAIPA Automotive Company), and Fameli (Iran National Copper Industries).

The data spans from October 22, 2018, to October 22, 2025, with daily closing prices recorded for each trading day. This results in a comprehensive time series dataset with 1,678 observations for each asset, providing a substantial historical record for analysis and modeling.

To facilitate the predictive modeling task, the dataset is partitioned such that the last 90 days of data (from July 2025 to October 2025) are reserved as the test set for evaluating the accuracy of the forecasts. The remaining data (from October 2018 to June 2025) is used for training the deep learning models, such as CNN and GRU, to capture the underlying patterns and dependencies in the price movements.

The objective of this case study is to develop a predictive model capable of forecasting the closing prices for each of the 15 assets over a future horizon of 90 days, leveraging the historical price trends and inter-asset relationships present in the dataset. The preprocessing and filtering described above for Case Study I were also applied to Case Study II.

## 4 Bootstrap-based price prediction

Following the justification provided in the previous sections, this paper employs CNN and GRU models to predict asset returns, in each case study.

**Case Study I.**

The CNN architecture consists of 64 one-dimensional convolutional kernels of size 3 in the first layer, using the ReLU activation function, followed by a pooling layer of size 2, and a final dense layer for prediction. The GRU architecture comprises 32 GRU units with the ReLU activation function, followed by a dense layer for prediction.

**Fig 1:**
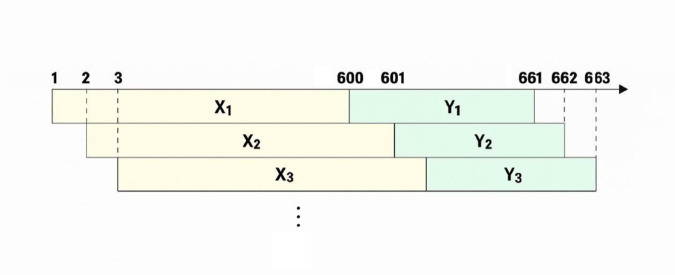
Training sample extraction via sliding window.

The nature of this problem dictates a supervised learning framework, thereby mandating the use of data with corresponding output labels for training the models. Accordingly, the training dataset was constructed such that each input sample, *x*-train, consists of a sequence of returns over 600 days, while its corresponding target, *y*-train, is the return 60 days into the future. Fig 1 demonstrates how the training samples are constructed. Therefore, for the task of predicting the returns of the final 60 days, which were excluded from the training set, the returns from the 600-day window leading up to this final period are utilized as the test data.

During training, the proposed CNN and GRU models were fitted to the dataset (*x*-train, *y*-train) using 100 epochs and a batch size of 50. The data sequence was kept in order (shuffle=False) to preserve temporal dependencies within the time series data. The model parameters were optimized using the Adam optimizer, and the MSE was employed as the loss function to minimize the squared difference between predicted and actual values. The MAE metric was also monitored throughout training to evaluate predictive accuracy.

To model uncertainty in the predictions, we employed the bootstrapping technique. We generated 15 bootstrap samples by resampling the original training data with replacement. Each of these samples was subsequently used to train an instance of our predictor models. This provides 15 different prediction trajectories, which we use as 15 different scenarios in our scenario optimization task. The effect of the number of Bootstrap samples on the final optimization results is investigated in Case study II (see Fig 13). Note that our approach of first creating windows and then applying bootstrap resampling is statistically equivalent to block bootstrapping within our specific data preprocessing framework. The core principle of both methods is identical: to preserve the short-term temporal dependencies within contiguous blocks of data (which correspond exactly to our network input windows) while resampling these units to generate new training realizations. Whether one resamples blocks from the raw series and then creates windows, or creates windows first and then resamples them, the fundamental outcome is the same: the model is trained on a set of sequences where the critical intra-window dynamics are fully intact, and the resampling process provides the uncertainty modeling benefits of bootstrapping. Our method offers a practical and computationally efficient implementation of this principle, directly bootstrapping the (input, target) pairs that the CNN or GRU model will process. This is functionally analogous to block bootstrapping with a block length equal to our window size, as it preserves the same joint distribution of input sequences and their subsequent targets. The discontinuities introduced between resampled windows are a feature, not a bug, mirroring the block boundaries in traditional block bootstrapping and serving the same statistical purpose. Therefore, the proposed approach robustly fulfills the objective of evaluating model performance under resampling while being ideally suited for the sequence-based architecture of our models.

**Fig 2:**
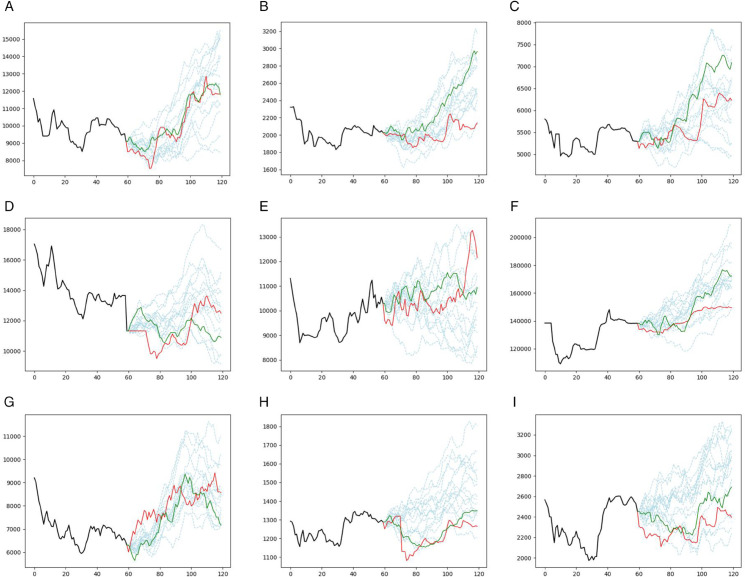
Prediction results for the CNN model (Case study I): Akhaber (top left), Asia (top middle), Foulad (top right), Hkshti (middle left), Mafakher (center), Nori (middle right), Satran (bottom left), Shasta (bottom middle), and Tejarat (bottom right). The different prediction scenarios are shown as light blue dashed lines, the single prediction trajectory without bootstrapping is shown as a green solid line, and the actual prices are shown as a red solid line. The horizontal axis denotes the number of days, and the vertical axis denotes the asset prices.

**Fig 3:**
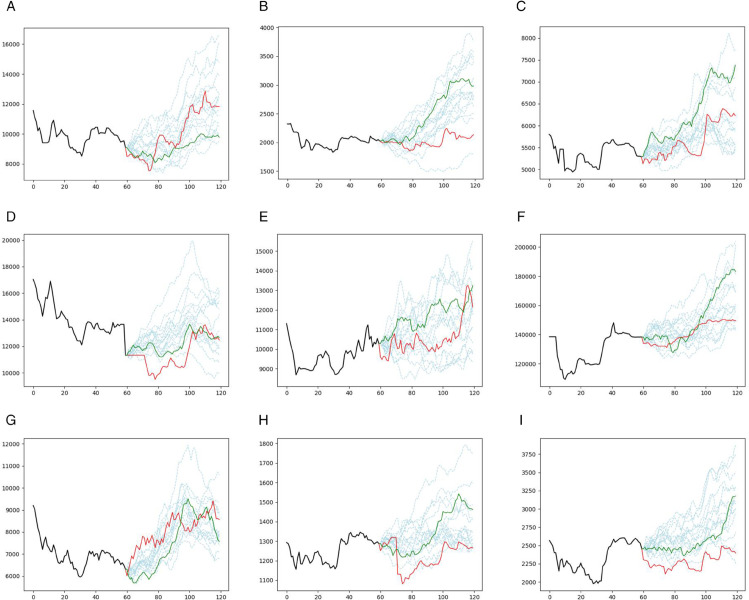
Prediction results for the GRU model (Case study I): Akhaber (top left), Asia (top middle), Foulad (top right), Hkshti (middle left), Mafakher (center), Nori (middle right), Satran (bottom left), Shasta (bottom middle), Tejarat (bottom right). The different prediction scenarios are plotted in light blue dashed lines, the single prediction trajectory without bootstrapping is plotted in a green solid line, and the real prices are also plotted in a red solid line. The horizontal axis denotes the number of days, and the vertical axis denotes the asset prices.

Fig 2, presents different prediction trajectories for the CNN model and all assets. The different prediction scenarios are plotted in light blue dashed lines, while the single prediction trajectory of the CNN model without bootstrapping is plotted in a green solid line. The real prices are also plotted in a red solid line. The corresponding plot of the GRU model is shown in Fig 3.

Finally, to assess the prediction accuracy of the models, we have utilized the MSE and Mean Absolute Error (MAE) metrics. Tables 3 and 4 display these two metrics for the predictions derived by the CNN model. As a result of our bootstrapping methodology, 15 distinct prediction trajectories are generated for each asset. Consequently, we have obtained 15 corresponding values for each performance metric (MSE and MAE). For each asset, Table 3 reports the average of these 15 values, with the standard deviation shown in parentheses to indicate the variability of the metrics. The corresponding tables for the GRU model are shown in Tables 5 and 6. As evidenced by the results in Tables 3 and 5, when the bootstrapping approach is implemented on the data, the CNN model yields lower MSE and MAE values compared to the GRU model, indicating superior predictive performance in this case. Conversely, in the absence of bootstrapping (as shown in Tables 4 and 6), the GRU model generally achieves lower MSE and MAE values in most assets, demonstrating better performance under these conditions.

**Table 3:** MSE and MAE means (standard deviation) with bootstrapping for the CNN model - Case study I.

Asset	MSE	MAE
Akhaber	0.0011 (0.0001)	0.0260 (0.0015)
Asia	0.0010 (9.9e-05)	0.0251 (0.0017)
Foulad	0.0011 (7.7e-05)	0.0247 (0.0014)
Hkshti	0.0011 (0.0002)	0.0256 (0.0020)
Mafakher	0.0012 (0.0002)	0.0272 (0.0021)
Nori	0.0010 (9.7e-05)	0.0244 (0.0016)
Satran	0.0011 (0.0002)	0.0257 (0.0028)
Shasta	0.0009 (7.9e-05)	0.0235 (0.0011)
Tejarat	0.0010 (0.0001)	0.0252 (0.0013)

**Table 4:** MSE and MAE without bootstrapping for the CNN model - Case study I.

	Akhaber	Asia	Foulad	Hkshti	Mafakher	Nori	Satran	Shasta	Tejarat
MSE	0.0011	0.0012	0.0011	0.0013	0.0010	0.0011	0.0014	0.0009	0.0011
MAE	0.0245	0.0266	0.0251	0.0278	0.0250	0.0254	0.0275	0.0224	0.0247

**Table 5:** MSE and MAE means (standard deviation) with bootstrapping for the GRU model - Case study I.

Asset	MSE	MAE
Akhaber	0.0011 (0.0001)	0.0263 (0.0023)
Asia	0.0011 (0.0002)	0.0253 (0.0022)
Foulad	0.0011 (0.0001)	0.0251 (0.0019)
Hkshti	0.0012 (0.0002)	0.0263 (0.0016)
Mafakher	0.0013 (0.0002)	0.0283 (0.0021)
Nori	0.0010 (0.0001)	0.0245 (0.0019)
Satran	0.0012 (0.0001)	0.0270 (0.0019)
Shasta	0.0010 (7.5e-05)	0.0239 (0.0014)
Tejarat	0.0010 (0.0002)	0.0260 (0.0028)

**Table 6:** MSE and MAE without bootstrapping for the GRU model - Case study I.

	Akhaber	Asia	Foulad	Hkshti	Mafakher	Nori	Satran	Shasta	Tejarat
MSE	0.0011	0.0013	0.0009	0.0012	0.0014	0.0010	0.0009	0.0008	0.0012
MAE	0.0262	0.0263	0.0231	0.0270	0.0274	0.0252	0.0241	0.0227	0.0266

### Case Study II.

Following the methodology outlined for Case Study I, we further evaluate the performance of CNN and GRU models for asset return prediction. The architectures of the CNN and GRU networks in this case study have been slightly modified. The CNN architecture consists of a one-dimensional convolutional layer with 8 filters, a kernel size of 5, and a ReLU activation function. The output of this layer is reshaped and passed through a two-dimensional convolutional layer with 4 filters, a kernel size of 8×3, and a ReLU activation function. The network then applies two-dimensional max-pooling and a flattening operation, followed by a dropout layer (rate = 0.1) to reduce overfitting. A final dense layer with 90 neurons produces predictions for the next 90 days. The GRU architecture comprises 64 GRU units with the tanh activation function, followed by a dense layer with 90 neurons for prediction.

The training procedure maintained the same parameters: 100 epochs with a batch size of 100, preserving temporal order (shuffle = False) to respect time series dependencies. The Adam optimizer with a learning rate of $5\;\times \;10^{-4}$ was employed for both models, utilizing the Huber loss function to balance sensitivity to small errors and robustness against outliers, while monitoring MSE as the primary accuracy metric.

**Fig 4:**
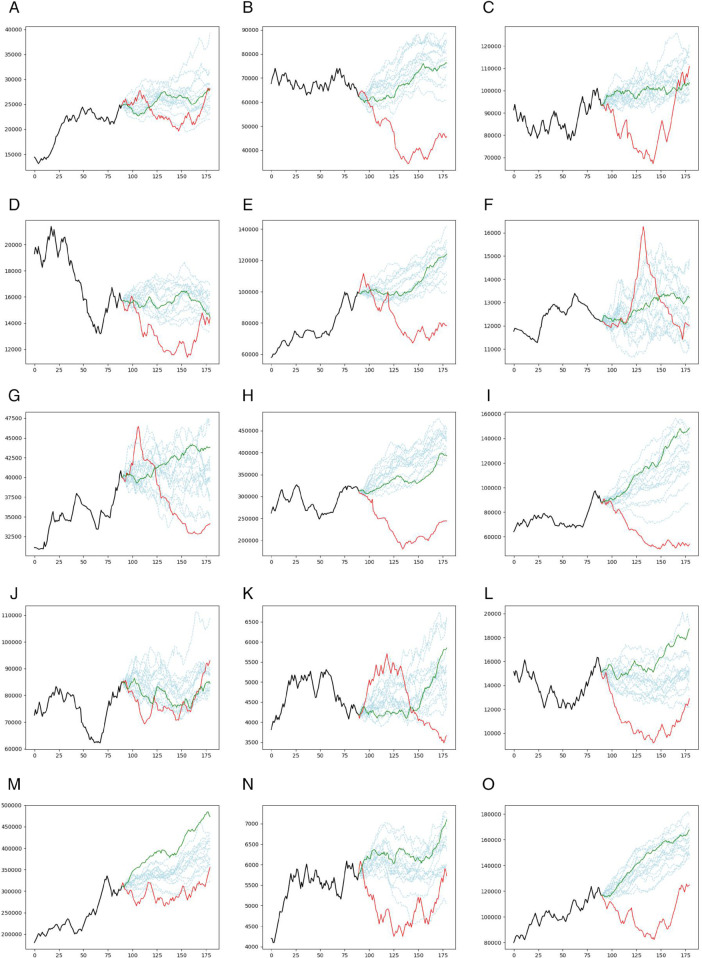
**Prediction results of the CNN model in Case Study II for 15 assets:** prediction scenarios (light-blue dashed), single-step prediction (green solid), and actual prices (red solid).

**Fig 5:**
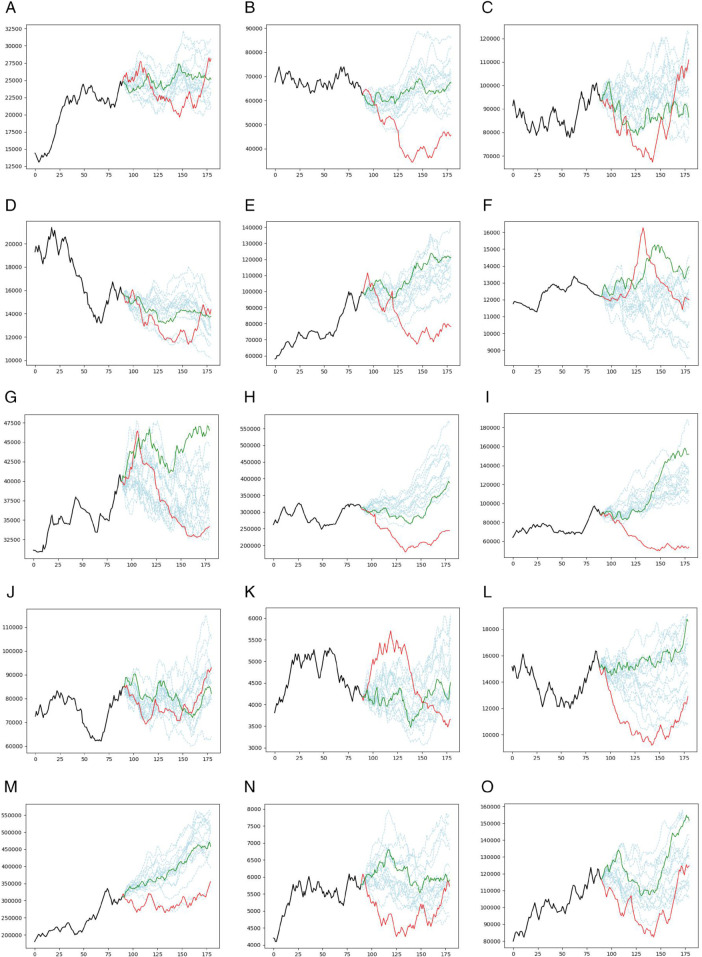
**Prediction results for the GRU model in Case Study II for 15 assets:** prediction scenarios (light blue dashed), single prediction (green solid), actual prices (red solid).

Fig 4 illustrates the prediction trajectories generated by the CNN model across all 15 assets. The light blue dashed lines represent various prediction scenarios, while the solid green line denotes the single prediction trajectory without bootstrapping. Actual prices are shown in solid red lines. Correspondingly, Fig 5 displays the prediction results for the GRU model under the same configuration.

To quantitatively assess model performance, we computed MSE and MAE metrics for both architectures without bootstrapping. Tables 8 and 10 present these metrics for the CNN and GRU models respectively across all 15 assets. The results indicate that both models achieve comparable performance across most assets, with the GRU model showing slightly better performance on certain assets while the CNN performs better on others. As shown in Tables 7–10, both with and without the bootstrap procedure, the CNN model yields lower MSE and MAE values than the GRU model for most assets, indicating its superior prediction performance.

**Table 7:** MSE and MAE means (standard deviation) with bootstrapping for the CNN model - Case study II.

Asset	MSE	MAE
Khodro	0.0256 (0.0275)	2.0052 (0.0792)
Foulad	0.2653 (0.0987)	1.9538 (0.0947)
Shepna	0.0112 (0.0089)	1.9498 (0.0878)
Kgl	0.0055 (0.0068)	1.5088 (0.0790)
Sefars	0.01857 (0.0689)	1.7985 (0.0721)
Drouz	0.0089 (0.0121)	1.1566 (0.0864)
Bswitch	0.0335 (0.0308)	1.0537 (0.0990)
Kkhak	0.2923 (0.0558)	1.4906 (0.1144)
Hkshti	0.6733 (0.2048)	1.8959 (0.1004)
Ghshasfa	0.0112 (0.0091)	1.3761 (0.1297)
Sakht	0.1543 (0.1026)	1.8008 (0.1295)
Ghsabet	0.0288 (0.0328)	1.6582 (0.1310)
Sesoufi	0.0114 (0.0106)	1.7912 (0.0636)
Khsapa	0.0139 (0.0137)	1.9121 (0.0754)
Fameli	0.0518 (0.0274)	1.7332 (0.0833)

**Table 8:** MSE and MAE without bootstrapping for the CNN model - Case study II.

Asset	Khodro	Foulad	Shepna	Kgl	Sefars	Drouz	Bswitch	Kkhak
MSE	0.0485	0.0602	0.0486	0.0344	0.0513	0.0167	0.0135	0.0331
MAE	1.8606	1.8260	1.7222	1.4312	1.7874	0.8905	0.7895	1.2515
**Asset**	**Hkshti**	**Ghshasfa**	**Sakht**	**Ghsabet**	**Sesoufi**	**Khaspa**	**Fameli**	
MSE	0.0548	0.0227	0.0512	0.0433	0.0575	0.0466	0.0425	
MAE	1.9062	1.1313	1.8615	1.6554	1.9979	1.8401	1.6787	

**Table 9:** MSE and MAE means (standard deviation) with bootstrapping for the GRU model - Case study II.

Asset	MSE	MAE
Khodro	0.0297 (0.0302)	2.1144 (0.1383)
Foulad	0.1676 (0.0993)	2.0698 (0.1394)
Shepna	0.0367 (0.0431)	2.0877 (0.0864)
Kgl	0.0301 (0.0326)	1.5744 (0.1057)
Sefars	0.1398 (0.0660)	1.9900 (0.0891)
Drouz	0.0244 (0.0315)	1.4081 (0.0952)
Bswitch	0.0197 (0.0219)	1.2324 (0.1187)
Kkhak	0.3073 (0.1406)	1.5575 (0.0721)
Hkshti	0.6710 (0.2605)	2.0954 (0.1661)
Ghshasfa	0.0204 (0.0326)	1.6015 (0.1289)
Sakht	0.0870 (0.0789)	2.0260 (0.1312)
Ghsabet	0.0545 (0.0520)	1.8333 (0.0958)
Sesoufi	0.0911 (0.0526)	1.9479 (0.1118)
Khsapa	0.0197 (0.0248)	2.0184 (0.1049)
Fameli	0.0132 (0.0132)	1.7785 (0.0887)

**Table 10:** MSE and MAE without bootstrapping for the GRU model - Case study II.

Asset	Khodro	Foulad	Shepna	Kgl	Sefars	Drouz	Bswitch	Kkhak
MSE	0.0555	0.0761	0.0876	0.0384	0.0630	0.0326	0.0219	0.0451
MAE	1.8702	2.0479	2.3081	1.5990	2.0088	1.3351	1.1488	1.6440
Asset	Hkshti	Ghshasfa	Sakht	Ghsabet	Sesoufi	Khaspa	Fameli	
MSE	0.0681	0.0351	0.0815	0.0528	0.0573	0.0657	0.0520	
MAE	2.0391	1.3994	2.2406	1.8494	1.8866	2.0654	1.8713	

## 5 Scenario-based portfolio optimization

This section is devoted to a scenario-based modelling for portfolio construction. Sect 5.1 examines the process of constructing the tracking model for integrating the optimal solutions obtained from solving the portfolio optimization problem for each scenario. Sect 5.2 introduces the Portfolio Deviation (PD) criterion and compares the optimal solutions derived from the tracking model with those obtained from the MV model without bootstrapping.

### 5.1 Portfolio construction

The predicted returns of the 60 days are used to estimate the means, variances and covariances, which are used for portfolio optimization based on the MV model [1]. For each of the 15 prediction scenarios, different parameters were obtained for the portfolio. Thus, we introduce the following scenario-based portfolio optimization problem. Let for scenario s∈S, $\mu_{s}$ represent the estimated expected return, $\Sigma_{s}$ denote the estimated variance-covariance matrix, and *λ* is the risk aversion coefficient. Then a combination of problems (2) and (7) would be introduced as follows:

$$\forall s\in S:\;\min_{x}\;\lambda x^{T}\Sigma_{s}x-(1-\lambda )\mu_{s}^{T}x\;\;\;\;\;\;\;\;s.t.\;\;\;\;e^{T}x=1,\;\;\;\;\;\;\;\;\;x\geq 0.\;$$ (9)

For each scenario s∈S, let ${\hat{x}}_{s}$ be an optimal solution of problem (9), and ${\hat{\nu }}_{s}$ be the optimal value. Then the tracking model for problem (9) is as follows:

$$\min\;\sum_{s\in S}P_{s}d(\lambda x^{T}\Sigma_{s}x-(1-\lambda )\mu_{s}^{T}x,{\hat{\nu }}_{s}),\;\;s.t.\;\;\;\;e^{T}x=1,\;\;\;\;\;\;\;\;\;\;x\geq 0,\;$$ (10)

where *P*_*s*_ is the probability of scenario *s* and *d* is a distance measure. Since the probability of all bootstrap samples is equal, we assign $P_{s}=\frac{1}{15}$ for each s∈S. Furthermore, we define *d* as the squared distance measure, leading to the following mathematical programming problem:

$$\min\;\frac{1}{15}\sum_{s=1}^{15}(\lambda x^{T}\Sigma_{s}x-(1-\lambda )\mu_{s}^{T}x-{\hat{\nu }}_{s}{)}^{2},\;\;s.t.\;\;\;\;e^{T}x=1,\;\;\;\;\;\;\;\;\;x\geq 0.\;$$ (11)

**Fig 6:**
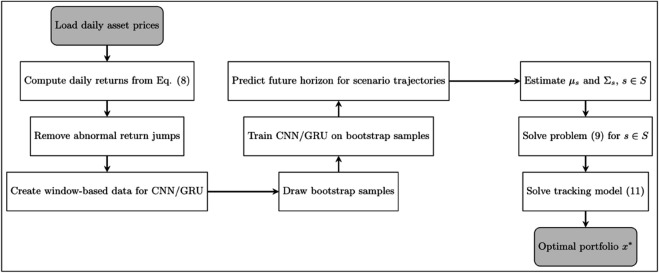
Flowchart of the scenario-based portfolio optimization.

Fig 6 depicts the flowchart of our approach. A detailed description of each box (stage) has thus far been provided in the manuscript text.

As can be inferred from Theorem 1 below, when $\Sigma_{s},\;\;s\in S,$ is positive semi-definite, problem (11) is a tractable convex programming problem which can be efficiently solved by using convex optimization tools [53].

**Theorem 1.** If $\Sigma_{s},\;s\in S,$ is positive semi-definite, then Problem (11) is a convex programming problem.

**Proof.** Assume $\Sigma_{s},\;\;s\in S,$ is positive semi-definite. Set $f_{s}(x):=(\lambda x^{T}\Sigma_{s}x\;-\;(1\;-\;\lambda )\mu_{s}^{T}x\;-\;{\hat{\nu }}_{s}{)}^{2}$. We should prove that the objective function of (11) is convex over the feasible set of (11), which is $𝒳:=\{x\in {\mathbb{R}}^{N}:\;e^{T}x=1,\;x\geq 0\}.$ To this end, it is sufficient to show that *f*_*s*_(*x*) is convex over 𝒳. We have $f_{s}(x)=(g_{s}(x){)}^{2}$ where $g_{s}(x):=(\lambda x^{T}\Sigma_{s}x-(1-\lambda )\mu_{s}^{T}x-{\hat{\nu }}_{s})$. Since $\Sigma_{s}$ is positive semi-definite, the Hessian matrix of the quadratic function *g*_*s*_(*x*) is positive semi-definite, and hence it is convex. Furthermore, *g*_*s*_(*x*) is nonnegative over 𝒳, because ${\hat{\nu }}_{s}$ is the minimum of $\left(\lambda x^{T}\Sigma_{s}x-(1-\lambda )\mu_{s}^{T}x\right)$ over 𝒳. Now, considering x,y∈𝒳 and λ∈[0,1], we have λx+(1−λ)y∈𝒳 and $0\leq g_{s}(\lambda x+(1-\lambda )y)\leq \lambda g_{s}(x)+(1-\lambda )g_{s}(y)$, leading to


$$(g_{s}(\lambda x+(1-\lambda )y){)}^{2}\leq (\lambda g_{s}(x)+(1-\lambda )g_{s}(y){)}^{2}.$$

Since the univariate function *h*(*t*) = *t*^2^ is convex over [0,+∞), we get


$$\begin{array}{cc} (\lambda g_{s}(x)+(1-\lambda )g_{s}(y){)}^{2} & =h(\lambda g_{s}(x)+(1-\lambda )g_{s}(y))\\ & \leq \lambda h(g_{s}(x))+(1-\lambda )h(g_{s}(y))\\ & =\lambda (g_{s}(x){)}^{2}+(1-\lambda )(g_{s}(y){)}^{2}. \end{array}$$

Therefore,


$$f_{s}(\lambda x+(1-\lambda )y)=(g_{s}(\lambda x+(1-\lambda )y){)}^{2}\leq \lambda (g_{s}(x){)}^{2}+(1-\lambda )(g_{s}(y){)}^{2}=\lambda f_{s}(x)+(1-\lambda )f_{s}(y).$$

It means *f*_*s*_(*x*) is convex over 𝒳, and the proof is complete. □

Tables 15–50 in Appendix B.1 report the optimal solutions, corresponding risks, and returns derived from the tracking model, the MV problem without bootstrapping, and the MV problem using real data for return values derived from CNN and GRU models, across varying levels of the risk aversion parameter *λ*, ranging from 0.1 to 0.9. As evident in these tables, the portfolio constructed by the tracking model exhibits greater diversification. This characteristic is highly desirable from a capital management perspective. In contrast, under MV model without bootstrapping, in most cases, the entire investment is allocated to a single asset. Moreover, the selected asset often differs from the one chosen by the MV model when using the real data. This lack of diversification leads to suboptimal performance for the MV model without bootstrapping.

It is worth mentioning that the main focus of the analysis in this study is based on the portfolio deviation notion and the Pareto front, which will be introduced and discussed in detail in the next subsection. Therefore, the data presented in the aforementioned tables do not play a significant role in the main findings of our study; accordingly, their reporting has been limited to the appendix.

### 5.2 Analysis

In this section, the implementation results of the tracking model (11) are presented for return values derived from CNN and GRU models, for $\lambda =\frac{i}{10},\;i=1,2,\ldots ,9$. To compare the performance of the tracking model (11) based on the MV model (6) (equivalently, (7)) with predicted returns without bootstrapping, we use the Portfolio Deviation (PD) criterion defined as


$$PD=\sum_{i=1}^{9}|{\hat{x}}_{i}-x_{i}|$$

where ${\hat{x}}_{i}$ is the solution of each of the competitor models (11) and (6) for asset *i* and *x*_*i*_ represents the solution of (6) using the true return values.

#### Case Study I.

Tables 11 and 12 present the PD values based on the results obtained from the CNN and GRU models, respectively. As it can be observed from these tables, the portfolio constructed using the tracking model (11) consistently exhibits smaller PD values than model (6) without bootstrapping. This empirical finding highlights the effectiveness of the tracking model in aligning predicted portfolio performance with real market behavior, thereby offering a more reliable framework for practical portfolio construction. By dividing the PD values for model (6) without bootstrapping by those of the tracking model across different values of *λ*, the PD ratios are obtained, which are reported in the last column of Tables 11 and 12 for the CNN and GRU models, respectively. The last row reports the average of the corresponding column. In fact, the larger these ratios are, the greater the discrepancy between the PD values of the non-bootstrapped model (6) and those of the tracking model.

**Table 11:** Portfolio Deviation for CNN model - Case study I. Lower *λ* values emphasize higher expected returns at greater risk levels, whereas larger *λ* values prioritize risk minimization and yield more conservative portfolios.

λ	Tracking model	Without bootstrapping	PD ratio
0.1	**1.369**	2.000	1.461
0.2	**1.411**	2.000	1.417
0.3	**1.173**	2.000	1.705
0.4	**1.354**	2.000	1.477
0.5	**1.083**	2.000	1.847
0.6	**0.905**	2.000	2.210
0.7	**1.212**	2.000	1.650
0.8	**1.376**	2.000	1.453
0.9	**1.463**	1.906	1.303
Average			1.614

**Table 12:** Portfolio Deviation for GRU model - Case study I. Lower *λ* values emphasize higher expected returns at greater risk levels, whereas larger *λ* values prioritize risk minimization and yield more conservative portfolios.

λ	Tracking model	Without bootstrapping	PD ratio
0.1	**1.904**	2.000	1.050
0.2	**1.936**	2.000	1.033
0.3	**1.950**	2.000	1.026
0.4	**1.904**	2.000	1.050
0.5	**1.638**	2.000	1.221
0.6	**1.433**	2.000	1.396
0.7	**1.371**	2.000	1.459
0.8	**1.447**	2.000	1.382
0.9	**1.463**	1.906	1.303
Average			1.213

As shown in Tables 11 and 12, the PD of portfolios generated by the tracking model (11) is consistently smaller, across all values of the risk aversion parameter *λ*, compared to the PD values obtained from model (6) without bootstrapping.

**Fig 7:**
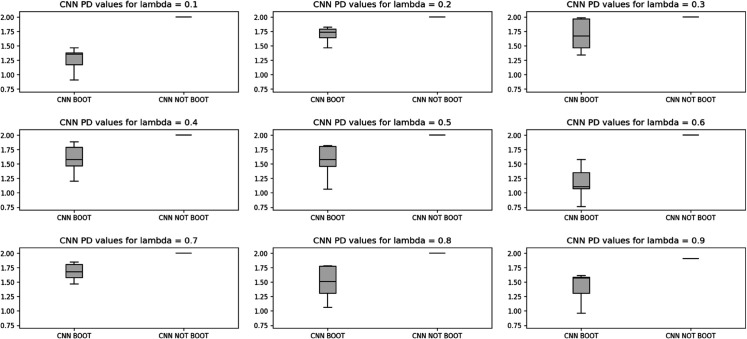
Box plot of Portfolio Deviation values obtained from 10 replications of the tracking model using CNN model - Case study I.

**Fig 8:**
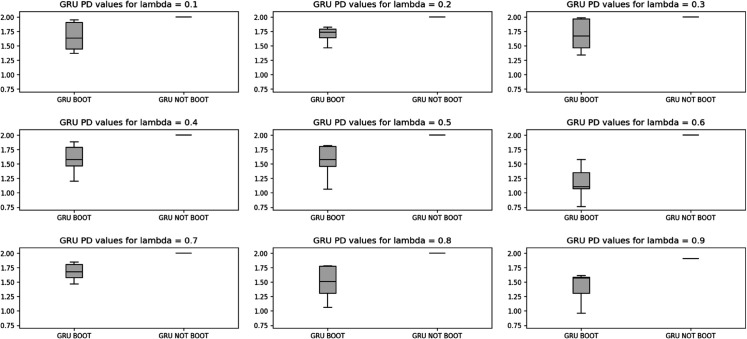
Box plot of Portfolio Deviation values obtained from 10 replications of the tracking model using GRU model - Case study I.

To study the effect of randomness of bootstrap sample generation on the PD values, and to perform a sensitivity analysis, 10 independent replications were conducted, each generating separate 15 bootstrap scenarios. Figs 7 and 8, present the box plots of PD values across 10 replications, for CNN and GRU models, respectively, which fall completely lower than the PD obtained from Model (6) without bootstrapping. This result indicates the superior capability of the tracking model in producing portfolios that are more closely aligned with true market outcomes.

**Fig 9:**
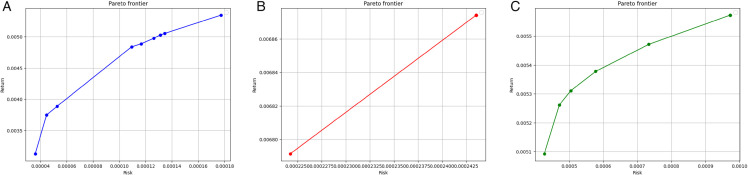
Pareto frontiers for the CNN model - Case study I: The tracking model (11) (left), model (6) without bootstrapping (middle), and model (6) with true return values (right). The horizontal axis denotes risk, and the vertical axis denotes return.

**Fig 10:**
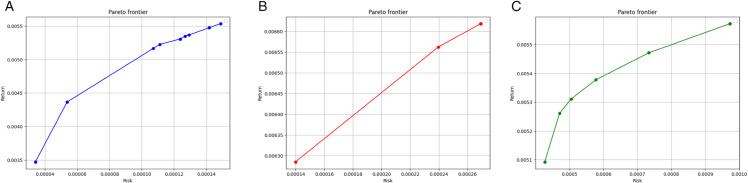
Pareto frontiers for the GRU model - Case study I: The tracking model (11) (left), model (6) without bootstrapping (middle), and model (6) with true return values (right). The horizontal axis denotes risk, and the vertical axis denotes return.

Figs 9 and 10 illustrate the Pareto frontiers derived from the tracking model (11), model (6) without bootstrapping, and model (6) applied to the true return values, corresponding to both CNN and GRU prediction models, respectively. As can be observed, the Pareto frontier generated by the tracking model is consistently closer to the frontier constructed using real data, demonstrating its superior capability in replicating actual portfolio behavior compared to the model without bootstrapping.

#### Case Study II.

Tables 13 and 14 present the PD values based on the results obtained from the CNN and GRU models for Case Study II, respectively. Consistent with the findings in Case Study I, the portfolio constructed using the tracking model (11) exhibits smaller PD values than model (6) without bootstrapping across most values of *λ*. This further validates the effectiveness of the tracking model in aligning predicted portfolio performance with real market behavior, providing a more reliable framework for practical portfolio construction.

**Table 13:** Portfolio Deviation for CNN model - Case study II. Lower *λ* values emphasize higher expected returns at greater risk levels, whereas larger*λ* values prioritize risk minimization and yield more conservative portfolios.

λ	Tracking model	Without bootstrapping	PD ratio
0.1	**1.8667**	1.999994	1.071
0.2	**1.8667**	1.999993	1.071
0.3	**1.8661**	1.999992	1.072
0.4	**1.7332**	1.999996	1.154
0.5	**1.6084**	1.999996	1.243
0.6	**1.5998**	1.999988	1.250
0.7	**1.5733**	1.999994	1.271
0.8	**1.4654**	1.999990	1.365
0.9	**1.3327**	1.991399	1.494
Average			1.221

**Table 14:** Portfolio Deviation for GRU model - Case study II. Lower *λ* values emphasize higher expected returns at greater risk levels, whereas larger *λ* values prioritize risk minimization and yield more conservative portfolios.

*λ*	Tracking model	Without bootstrapping	PD ratio
0.1	**1.9812**	1.999994	1.009
0.2	**1.9808**	1.999992	1.010
0.3	**1.9781**	1.999992	1.011
0.4	**1.9466**	1.999996	1.027
0.5	**1.8058**	1.999996	1.107
0.6	**1.5998**	1.999987	1.250
0.7	**1.5733**	1.999939	1.271
0.8	**1.4654**	1.635181	1.116
0.9	**1.3327**	1.722062	1.292
Average			1.121

By dividing the PD values for model (6) without bootstrapping by those of the tracking model across different values of *λ*, the PD ratios are obtained, which are reported in the last column of Tables 13 and 14 for the CNN and GRU models, respectively. The last row reports the average of the corresponding column. The larger these ratios are, the greater the discrepancy between the PD values of the non-bootstrapped model and those of the tracking model.

As shown in Tables 13 and 14, the PD of portfolios generated by the tracking model (11) is consistently smaller, across all values of the risk aversion parameter *λ*, compared to the PD values obtained from model (6) without bootstrapping. The CNN model demonstrates higher average PD ratios (1.221) compared to the GRU model (1.121), indicating that the tracking model provides more substantial improvements over the non-bootstrapped approach when using CNN predictions in this case study.

**Fig 11:**
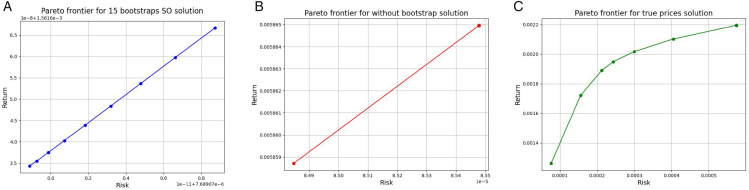
Pareto frontiers for the CNN model - Case study II: The tracking model (11) (left), model (6) without bootstrapping (middle), and model (6) with true return values (right). The horizontal axis denotes risk, and the vertical axis denotes return.

**Fig 12:**
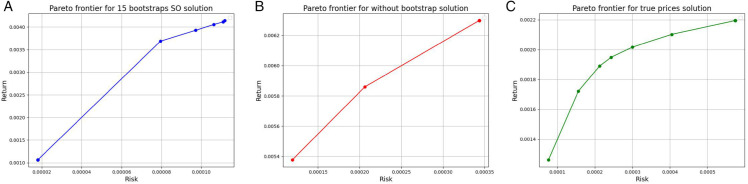
Pareto frontiers for the GRU model - Case study II: The tracking model (11) (left), model (6) without bootstrapping (middle), and model (6) with true return values (right), for the GRU model - Case study II. The horizontal axis denotes risk, and the vertical axis denotes return.

Figs 11 and 12 illustrate the Pareto frontiers derived from the tracking model (11), model (6) without bootstrapping, and model (6) applied to the true return values, corresponding to both CNN and GRU prediction models, respectively. As it can be observed, the Pareto frontier generated by the tracking model is consistently closer to the frontier constructed using real data, demonstrating its superior capability in replicating actual portfolio behavior compared to the model without bootstrapping.

**Fig 13:**
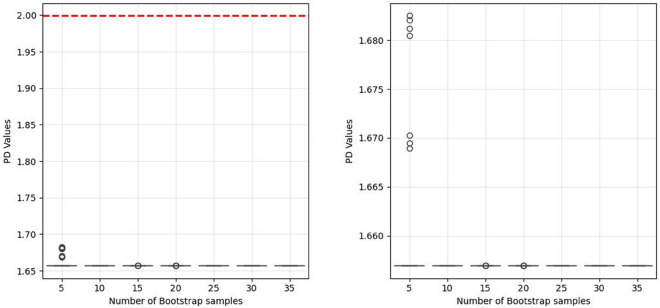
Sensitivity analysis of PD values for the tracking model with varying bootstrap samples.

To determine the appropriate number of bootstrap samples for the tracking model, we conducted a sensitivity analysis using different bootstrap sample sizes (5, 10, 15, 20, 25, 30, 35) with 50 random replications for each size. Fig 13 presents a comparative analysis of the average Portfolio Deviation (PD) values through boxplots, divided into two panels for detailed interpretation. The left panel displays the full range of PD values with the red dashed line representing the average PD value without bootstrapping. The right panel provides a zoomed-in view of the stabilized region, highlighting the subtle variations between 10 and 35 bootstrap samples. Each boxplot summarizes 50 random replications. Outliers, plotted as individual points, are more prevalent with 5 bootstrap samples, indicating higher variability and instability in PD values. The right panel provides a magnified view of the PD values for larger bootstrap samples (15-35), revealing that the performance gains plateau beyond 15 samples. For 5 bootstrap samples, the boxes have numerous outliers above the upper whiskers, reflecting considerable uncertainty and instability in the tracking model’s performance. As the number of bootstrap samples increases to 15 and beyond, the boxes have fewer outliers, indicating more consistent and reliable PD values. The red dashed line in the left panel, representing the average PD value without bootstrapping, lies well above all boxplots. This visual comparison underscores the superiority of the tracking model with adequate bootstrap sampling over the non-bootstrapped approach. Based on these observations, 10 to 15 bootstrap samples emerge as the optimal range, providing substantial performance improvement over the non-bootstrapped model while maintaining computational efficiency. The minimal gains beyond 15 samples do not justify the additional computational cost, making 15 bootstrap samples the recommended choice for our application.

## 6 Theoretical justification

In this section, we provide some results that highlight the theoretical aspects of our work and clarify its superiority over traditional methods such as worst-case robust optimization. To this end, we first present some preliminaries from multi-objective programming, briefly discussed below.

Multi-objective programming deals with optimization problems involving more than one objective function to be maximized or minimized simultaneously [48]. Such problems arise in many fields, including engineering, economics, health care, and portfolio optimization [20,48,54]. Let Ω be a nonempty subset of ${\mathbb{R}}^{n}$. Also, let $f:{\mathbb{R}}^{n}⟶{\mathbb{R}}^{p}$ be a vector-valued function defined as $f(x)=(f_{1}(x),f_{2}(x),\ldots ,f_{p}(x){)}^{T}$ for $x\in {\mathbb{R}}^{n}.$ Consider a general Multi-Objective Optimization Problem (MOP) as follows:

$$\begin{array}{cc} \min_{x} & f_{1}(x)\\ \min_{x} & f_{2}(x)\\ \;\vdots & \\ \min_{x} & f_{p}(x)\\ \;s.t. & x\in \Omega . \end{array}$$ (12)

Here, Ω denotes the set of feasible solutions of MOP (12), and $f_{1},f_{2},\ldots ,f_{p}$ represent the objective functions. In MOP, there is usually no solution x∈Ω that simultaneously optimizes all objective functions. Therefore, the concept of (weak) efficiency is used instead of classical optimality [48].

**Definition 1.** A feasible solution $x^{*}\in \Omega$ is called

- a *weakly efficient solution* to MOP (12) if there is no other x∈Ω such that $f_{i}(x)<f_{i}(x^{*})$ for all i=1,2,…,p.
- an *efficient solution* to MOP (12) if there is no other x∈Ω such that $f_{i}(x)\leq f_{i}(x^{*})$ for all i=1,2,…,p, and $f_{i}(x)<f_{i}(x^{*})$ for some i=1,2,…,p.

The sets of efficient and weakly efficient solutions of MOP (12) are denoted by $\Omega_{E}$ and $\Omega_{WE}$, respectively. It is well known that $\Omega_{E}\subseteq \Omega_{WE}$, and the quality of an efficient solution is better than that of weakly efficient solutions that are not efficient [48,55]. For example, consider a problem in ${\mathbb{R}}^{2}$ with two minimization objective functions, where


$$\Omega =\{A=(15,2),\;B=(5,2),\;C=(3,5),\;D=(5,5)\},\;f_{1}(x)=x_{1},\;f_{2}(x)=x_{2}.$$

In this problem, $\Omega_{E}=\{B,C\}$ and $\Omega_{WE}=\{A,B,C,D\}$. Here, *A* is a weakly efficient solution, although the value of the first objective function at this point is catastrophically poor. Indeed, weakly efficient solutions may perform well in some criteria but extremely poorly in others. Such points are not appropriate recommendations for users or decision makers. In particular, in investment problems, a portfolio corresponding to a weakly efficient solution of a portfolio optimization problem may have an extremely high investment risk or an undesirably low expected return.

Now, we examine our uncertain portfolio selection problem from a multi-objective optimization standpoint. Following Sect 5.1, for each scenario s∈S, $\mu_{s}$ represents the estimated expected return, $\Sigma_{s}$ denotes the estimated variance-covariance matrix, and *λ* is the risk-aversion coefficient. Assume that S={1,2,…,p}, i.e., we have *p* scenarios. In our case study, *p* = 15. Considering an objective function corresponding to each scenario, we arrive at the following MOP:

$$\begin{array}{cc} \min_{x} & \lambda x^{T}\Sigma_{1}x-(1-\lambda )\mu_{1}^{T}x\\ \min_{x} & \lambda x^{T}\Sigma_{2}x-(1-\lambda )\mu_{2}^{T}x\\ \;\vdots & \\ \min_{x} & \lambda x^{T}\Sigma_{p}x-(1-\lambda )\mu_{p}^{T}x\\ \text{s.t.} & e^{T}x=1,\\ & x\geq 0. \end{array}$$ (13)

Our final optimal portfolios are constructed by solving the aggregation problem (11). The following theorem shows that each optimal solution of this problem is an efficient solution of MOP (13). Theorem 2 can be derived from the results on compromise programming [48,56]; however, we provide a direct proof here for the sake of completeness.

**Theorem 2.** If x^*^ is an optimal solution to the aggregation Problem (11), then it is an efficient solution of MOP (13).

**Proof.** By indirect proof, assume that x^*^ is an optimal solution of (11), while it is not an efficient solution of MOP (13). Then, there exists some $x^{0}\in {\mathbb{R}}^{N}$ such that $e^{T}x^{0}=1,\;x^{0}\geq 0$ (some feasible portfolio) and


$$\left\{ \begin{array}{c} \lambda x^{T}\Sigma_{s}x^{0}-(1-\lambda )\mu_{s}^{T}x^{0}\leq \lambda x^{T}\Sigma_{s}x^{*}-(1-\lambda )\mu_{s}^{T}x^{*},\;\forall s\in S,\\ \lambda x^{T}\Sigma_{s}x^{0}-(1-\lambda )\mu_{s}^{T}x^{0}<\lambda x^{T}\Sigma_{s}x^{*}-(1-\lambda )\mu_{s}^{T}x^{*},\;\exists s\in S. \end{array}\right.$$

Hence,


$$\left\{ \begin{array}{c} 0\leq \lambda x^{T}\Sigma_{s}x^{0}-(1-\lambda )\mu_{s}^{T}x^{0}-{\hat{\nu }}_{s}\leq \lambda x^{T}\Sigma_{s}x^{*}-(1-\lambda )\mu_{s}^{T}x^{*}-{\hat{\nu }}_{s},\;\forall s\in S,\\ 0\leq \lambda x^{T}\Sigma_{s}x^{0}-(1-\lambda )\mu_{s}^{T}x^{0}-{\hat{\nu }}_{s}<\lambda x^{T}\Sigma_{s}x^{*}-(1-\lambda )\mu_{s}^{T}x^{*}-{\hat{\nu }}_{s},\;\exists s\in S. \end{array}\right.$$

This imply,


$$\sum_{s\in S}(\lambda x^{T}\Sigma_{s}x^{0}-(1-\lambda )\mu_{s}^{T}x^{0}-{\hat{\nu }}_{s}{)}^{2}<\sum_{s\in S}(\lambda x^{T}\Sigma_{s}x^{*}-(1-\lambda )\mu_{s}^{T}x^{*}-{\hat{\nu }}_{s}{)}^{2},$$

and contradicts the optimality of x^*^ for (11). The proof is complete. □

Now, we examine the handling of uncertainty in (Σ,μ) from a robust optimization standpoint. Here, we have *p* = 15 possible cases for these uncertain parameters. One of the most popular approaches for addressing this uncertainty is to construct an uncertainty set as


$$𝒰=\{(\Sigma_{s},\mu_{s}):\;s\in S\},$$

and then solve the following robust optimization problem:

$$\min_{x}\;\max_{(\Sigma ,\mu )\in 𝒰}\lambda x^{T}\Sigma x-(1-\lambda )\mu^{T}x\;\;\;s.t.\;\;\;\;e^{T}x=1,\;\;\;\;\;x\geq 0.\;$$ (14)

Theorem 3 below shows that each optimal solution of the robust optimization problem (14) is a weakly efficient solution of MOP (13). This theorem can also be derived from multi-objective programming scalarization theorems [48]; however, we provide a direct proof here for the sake of completeness.

**Theorem 3.** If x^*^ is an optimal solution to the robust optimization Problem (14), then it is a weakly efficient solution of MOP (13).

**Proof.** By indirect proof, assume that x^*^ is an optimal solution of (14), while it is not a weakly efficient solution of MOP (13). Then, there exists some $x^{0}\in {\mathbb{R}}^{N}$ such that $e^{T}x^{0}=1,\;x^{0}\geq 0$ (some feasible portfolio) and


$$\lambda x^{T}\Sigma_{s}x^{0}-(1-\lambda )\mu_{s}^{T}x^{0}<\lambda x^{T}\Sigma_{s}x^{*}-(1-\lambda )\mu_{s}^{T}x^{*},\;\forall s\in S.$$

These strict inequalities imply


$$\max_{s\in S}(\lambda x^{T}\Sigma_{s}x^{0}-(1-\lambda )\mu_{s}^{T}x^{0})<\max_{s\in S}(\lambda x^{T}\Sigma_{s}x^{*}-(1-\lambda )\mu_{s}^{T}x^{*}),$$

and contradicts the optimality of x^*^ for (14). The proof is complete. □

In summary, due to Theorems 2 and 3, handling the uncertainty of parameters (Σ,μ) by means of worst-case robust optimization leads to weakly efficient solutions, while addressing it through the aggregation problem (11) yields efficient solutions. On the other hand, as mentioned above and as well known in the literature [48,55], efficient solutions are of higher quality compared to merely weakly efficient ones. Weakly efficient solutions may perform well in some criteria but extremely poorly in others. The portfolios corresponding to such points are not appropriate recommendations for investors. In the problems under investigation, a feasible solution (portfolio) could be weakly efficient while minimizing $\lambda x^{T}\Sigma_{s}x\;-\;(1\;-\;\lambda )\mu_{s}^{T}x$ only for certain specific scenarios, without considering all possible scenarios. Moreover, the robust optimization problem (14) focuses on the worst-case scenario, and its optimal solutions may not perform well (in terms of achieving the desired objective) if the uncertain scenario that actually occurs differs from the worst-case one considered.

In addition to the advantages of our approach revealed through its implementation on real-world datasets in the previous sections, the discussions and results presented in this section highlight the superiority of the scenario-optimization-based aggregation model (11) compared with the robust optimization problem (14).

## 7 Conclusion

Portfolio construction is a critical issue in financial markets; however, financial data are subject to a variety of factors that introduce significant uncertainty. As a result, accurately forecasting asset returns presents a considerable challenge. Therefore, in this study, we investigated the simultaneous application of bootstrapping for modeling uncertainty, machine learning for price prediction, and scenario-based MV portfolio optimization to integrate predictive uncertainty into portfolio decisions. We employed CNN and GRU models for asset return prediction and used bootstrapping to generate multiple prediction scenarios. These scenarios facilitate the construction of a tracking model based on the MV optimization framework.

Empirical results using data from the Tehran Stock Market demonstrate that the proposed tracking model outperforms the standard MV model without bootstrapping. In the return prediction stage, the bootstrapped CNN model achieves lower MSE and MAE values than the non-bootstrapped alternative, further validating its superior performance. Consequently, portfolios constructed using the tracking model with CNN-based forecasts prove more desirable. In addition to illustrating the advantages of our approach by implementing it on real-world datasets, we theoretically proved that the resulting scenario optimization problem is a convex program that generates efficient solutions superior to those produced by worst-case robust optimization techniques.

Future research could explore alternative MV model formulations, other uncertainty modeling approaches and their integration with stochastic and/or scenario optimization frameworks. These directions are left for future work.

## Appendix

## A Convolutional Neural Networks

**Fig 14:**
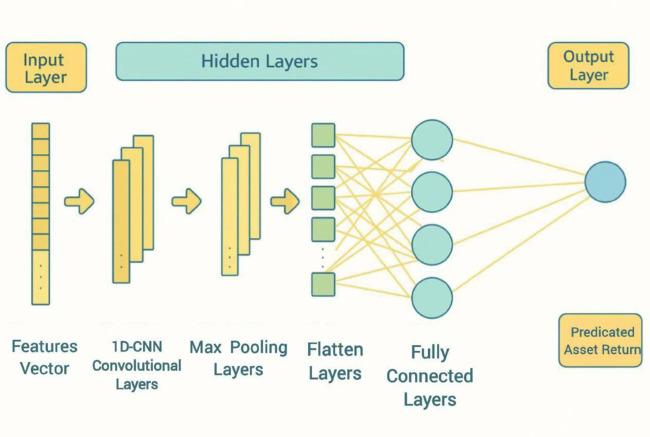
Architecture of a CNN (Adapted from [59, Fig 3]).

Convolutional Neural Networks (CNNs) represent one of the most powerful and widely used classes of deep learning models, especially in computer vision. A typical CNN architecture comprises two main components: a feature extractor that learns hierarchical representations from the input data, and a classifier or predictor that performs the final task, such as classification or regression [57]. While two and three-dimensional CNN architectures (2D-CNN and 3D-CNN) are the most relevant types of CNN in computer vision, the one-dimensional CNN (1D-CNN) is designed and introduced for sequential data and especially time series prediction [58]. Compared to the 2D-CNN architecture, 1D-CNN offers reduced computational complexity. The architecture of a typical 1D-CNN is depicted in Figs 14 (see also [59]). The main building blocks of a CNN consist of convolutional layers, pooling layers, fully connected layers, and activation functions [57,60].

**Convolutional layers:** The convolutional layer is one of the key components of a CNN, responsible for extracting features from the input data [57]. In a 1D-CNN structure, this layer consists of kernels that are one-dimensional vectors. Each kernel is responsible for extracting a distinct feature from the time series data, representing the dependence structure of the data. These kernels slide over the input vectors with a specified stride and perform the convolution operation.

**Pooling layers:** This layer essentially performs a down-sampling operation, reducing the dimensionality of the input data and consequently decreasing the number of connections in the network [61]. The primary objective of this layer is to lower computational complexity and prevent overfitting [62]. The most common types of pooling operations are average pooling and max pooling.

**Fully connected layers:** This layer is the last layer of the CNN, with each neuron connected to all neurons in the previous layer. The input to this layer is the output of the last pooling or convolutional layer, which is flattened before being passed to the next layer. The fully connected layer serves as the classifier or predictor within the network [57].

**Fig 15:**
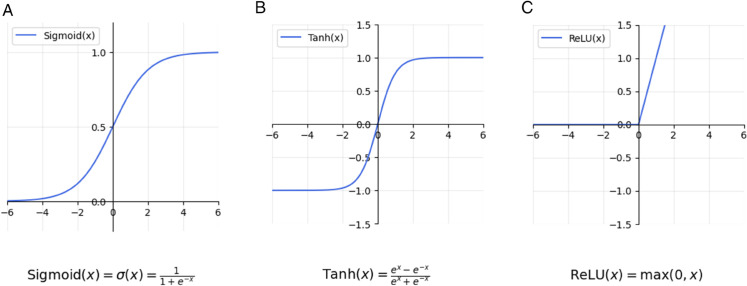
Graph and mathematical formulas of activation functions used in CNN.

**Activation functions:** Activation functions play a critical role in enabling CNNs to model complex nonlinear relationships within data. Without nonlinear activation functions, a CNN would be restricted to learning only simple linear mappings and would not be capable of accurately representing or capturing intricate and nonlinear patterns [63]. The most widely used nonlinear activation functions are ReLU, Tanh, and Sigmoid. The mathematical formulations and graphical representations of these functions are shown in Fig 15.

## B Gated Recurrent Unit

**Fig 16:**
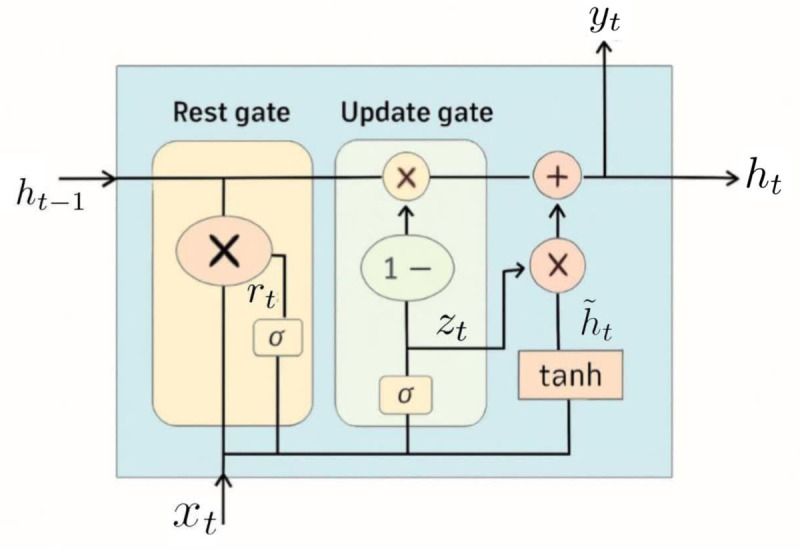
A cell of a GRU (Adapted from [65, Fig 2]).

The Gated Recurrent Unit (GRU) is a type of RNN that was inspired by the LSTM architecture. The GRU simplifies the LSTM architecture by merging two gates of the LSTM, which are input and forget gates, into a single update gate in its structure, leading to a more efficient design. A GRU cell comprises three core components: the update gate, the reset gate, and the current memory content [57]. These gating mechanisms allow the GRU to selectively retain and process information from prior time steps, effectively capturing long-term dependencies in time series data [64]. Fig 16 (see also [65]) illustrates the structure of a GRU unit.

**The update gate** determines how much of the past information should be retained at a given time step and combined with the current input [57]. This gate effectively addresses the vanishing gradient problem by enabling the model to learn what proportion of information should be carried forward [64]. Represented as *z*_*t*_ in the GRU structure, the output of an update gate is computed based on the previous hidden state *h*_*t*−1_ and the current input *x*_*t*_, as follows


$$z_{t}=\sigma \left(W_{z}[h_{t-1},x_{t}]+b_{z}\right),$$

where *W*_*z*_ is the vector of weights, *b*_*z*_ is the bias term, and σ(·) is the sigmoid activation function.

**The reset gate** determines how much of the past information should be forgotten. Denoted by *r*_*t*_, the output of this gate is similarly computed using different weights *W*_*r*_ and bias *b*_*r*_ as follows [57]:


$$r_{t}=\sigma \left(W_{r}[h_{t-1},x_{t}]+b_{r}\right).$$

**The current memory content** is calculated based on the outputs of reset, the previous hidden state, and the current input. A hyperbolic tangent activation function is then applied to this combination [57]:


$${\tilde{h}}_{t}=\text{tanh}\left(W_{h}[r_{t}h_{t-1},x_{t}]\right).$$

The current memory state *h*_*t*_ is updated by selection between *h*_*t*−1_ and ${\tilde{h}}_{t}$. The update gate *z*_*t*_ controls the balance between *h*_*t*−1_ and ${\tilde{h}}_{t}$. When *z*_*t*_ approaches zero, it indicates that the current memory content is less relevant, and the GRU unit tends to retain most of the information from the previous time step [64]:


$$h_{t}=\left(1-z_{t}\right)h_{t-1}+z_{t}{\tilde{h}}_{t}$$

Finally, the output *y*_*t*_ is calculated from the current memory state as follows [57]:


$$y_{t}=f\left(W_{c}h_{t}+b_{c}\right),$$

where f(·) is a suitable activation function, depending on the final task.

### B.1 Derived optimal solutions, risk, and return for CNN and GRU models

**Table 15:** Optimal solution, risk and return for the CNN model for λ=0.1 - Case study I.

	Tracking model	M-V without bootstrapping	M-V with real return data
Akhaber	**0.445**	0.000	0.000
Asia	**0.092**	**1.000**	0.000
Foulad	**0.020**	0.000	0.000
Hkshti	**0.017**	0.000	0.000
Mafakher	**0.013**	0.000	0.000
Nori	**0.045**	0.000	0.000
Satran	**0.315**	0.000	**1.000**
Shasta	**0.019**	0.000	0.000
Tejarat	**0.034**	0.000	0.000
Risk	1.3e-04	2.4e-04	9.7e-04
Return	5.0e-03	6.9e-03	5.6e-03

**Table 16:** Optimal solution, risk and return for the CNN model for λ=0.2 - Case study I.

	Tracking model	M-V without bootstrapping	M-V with real return data
Akhaber	**0.428**	0.000	0.000
Asia	**0.102**	**1.000**	0.000
Foulad	**0.024**	0.000	0.000
Hkshti	**0.019**	0.000	0.000
Mafakher	**0.015**	0.000	0.000
Nori	**0.053**	0.000	0.000
Satran	**0.295**	0.000	**1.000**
Shasta	**0.023**	0.000	0.000
Tejarat	**0.040**	0.000	0.000
Risk	1.2e-04	2.4e-04	9.7e-04
Return	4.9e-03	6.9e-03	5.6e-03

**Table 17:** Optimal solution, risk and return for the CNN model for λ=0.3 - Case study I.

	Tracking model	M-V without bootstrapping	M-V with real return data
Akhaber	**0.519**	0.000	0.000
Asia	**0.036**	**1.000**	0.000
Foulad	**0.004**	0.000	0.000
Hkshti	**0.004**	0.000	0.000
Mafakher	**0.002**	0.000	0.000
Nori	**0.010**	0.000	0.000
Satran	**0.413**	0.000	**1.000**
Shasta	**0.004**	0.000	0.000
Tejarat	**0.007**	0.000	0.000
Risk	1.8e-04	2.4e-04	9.7e-04
Return	5.3e-03	6.9e-03	5.6e-03

**Table 18:** Optimal solution, risk and return for the CNN model for λ=0.4 - Case study I.

	Tracking model	M-V without bootstrapping	M-V with real return data
Akhaber	**0.467**	0.000	0.000
Asia	**0.086**	**1.000**	0.000
Foulad	**0.018**	0.000	0.000
Hkshti	**0.014**	0.000	0.000
Mafakher	**0.009**	0.000	0.000
Nori	**0.039**	0.000	0.000
Satran	**0.323**	0.000	**1.000**
Shasta	**0.017**	0.000	0.000
Tejarat	**0.027**	0.000	0.000
Risk	1.3e-04	2.4e-04	9.7e-04
Return	5.0e-03	6.9e-03	5.6e-03

**Table 19:** Optimal solution, risk and return for the CNN model for λ=0.5 - Case study I.

	Tracking model	M-V without bootstrapping	M-V with real return data
Akhaber	**0.457**	0.000	**0.138**
Asia	**0.091**	**1.000**	0.000
Foulad	**0.019**	0.000	0.000
Hkshti	**0.015**	0.000	0.000
Mafakher	**0.009**	0.000	0.000
Nori	**0.041**	0.000	0.000
Satran	**0.320**	0.000	**0.862**
Shasta	**0.018**	0.000	0.000
Tejarat	**0.029**	0.000	0.000
Risk	1.3e-04	2.4e-04	7.3e-04
Return	5.0e-03	6.9e-03	5.5e-03

**Table 20:** Optimal solution, risk and return for the CNN model for λ=0.6 - Case study I.

	Tracking model	M-V without bootstrapping	M-V with real return data
Akhaber	**0.405**	0.000	**0.265**
Asia	**0.125**	**1.000**	0.000
Foulad	**0.026**	0.000	0.000
Hkshti	**0.020**	0.000	0.000
Mafakher	**0.015**	0.000	0.000
Nori	**0.061**	0.000	0.000
Satran	**0.282**	0.000	**0.735**
Shasta	**0.024**	0.000	0.000
Tejarat	**0.042**	0.000	0.000
Risk	1.1e-04	2.4e-04	5.8e-04
Return	4.9e-03	6.9e-03	5.4e-03

**Table 21:** Optimal solution, risk and return for the CNN model for λ=0.7 - Case study I.

	Tracking model	M-V without bootstrapping	M-V with real return data
Akhaber	**0.211**	0.000	**0.356**
Asia	**0.133**	**1.000**	0.000
Foulad	**0.064**	0.000	0.000
Hkshti	**0.062**	0.000	0.000
Mafakher	**0.066**	0.000	0.000
Nori	**0.118**	0.000	0.000
Satran	**0.183**	0.000	**0.644**
Shasta	**0.069**	0.000	0.000
Tejarat	**0.096**	0.000	0.000
Risk	5.3e-05	2.4e-04	5.0e-04
Return	3.9e-03	6.9e-03	5.3e-03

**Table 22:** Optimal solution, risk and return for the CNN model for λ=0.8 - Case study I.

	Tracking model	M-V without bootstrapping	M-V with real return data
Akhaber	**0.170**	0.000	**0.424**
Asia	**0.139**	**1.000**	0.000
Foulad	**0.096**	0.000	0.000
Hkshti	**0.075**	0.000	0.000
Mafakher	**0.037**	0.000	0.000
Nori	**0.136**	0.000	0.000
Satran	**0.142**	0.000	**0.576**
Shasta	**0.089**	0.000	0.000
Tejarat	**0.116**	0.000	0.000
Risk	4.5e-05	2.4e-04	4.7e-04
Return	3.7e-03	6.9e-03	5.3e-03

**Table 23:** Optimal solution, risk and return for the CNN model for λ=0.9 - Case study I.

	Tracking model	M-V without bootstrapping	M-V with real return data
Akhaber	**0.111**	0.000	**0.445**
Asia	**0.111**	**0.950**	0.000
Foulad	**0.117**	0.000	0.000
Hkshti	**0.111**	**0.050**	0.000
Mafakher	**0.111**	0.000	0.000
Nori	**0.117**	0.000	0.000
Satran	**0.100**	0.000	**0.498**
Shasta	**0.110**	0.000	0.000
Tejarat	**0.111**	0.000	0.000
Risk	3.6e-05	2.2e-04	4.3e-04
Return	3.1e-03	6.8e-03	5.1e-03

**Table 24:** Optimal solution, risk and return for the GRU model for λ=0.1 - Case study I.

	Tracking model	M-V without bootstrapping	M-V with real return data
Akhaber	**0.217**	0.000	0.000
Asia	**0.552**	**1.000**	0.000
Foulad	**0.022**	0.000	0.000
Hkshti	**0.021**	0.000	0.000
Mafakher	**0.020**	0.000	0.000
Nori	**0.023**	0.000	0.000
Satran	**0.048**	0.000	**1.000**
Shasta	**0.016**	0.000	0.000
Tejarat	**0.080**	0.000	0.000
Risk	1.2e-04	2.7e-04	9.7e-04
Return	5.3e-03	6.6e-03	5.6e-03

**Table 25:** Optimal solution, risk and return for the GRU model for λ=0.2 - Case study I.

	Tracking model	M-V without bootstrapping	M-V with real return data
Akhaber	**0.246**	0.000	0.000
Asia	**0.588**	**1.000**	0.000
Foulad	**0.014**	0.000	0.000
Hkshti	**0.013**	0.000	0.000
Mafakher	**0.011**	0.000	0.000
Nori	**0.014**	0.000	0.000
Satran	**0.032**	0.000	**1.000**
Shasta	**0.009**	0.000	0.000
Tejarat	**0.071**	0.000	0.000
Risk	1.4e-04	2.7e-04	9.7e-04
Return	5.5e-03	6.6e-03	5.6e-03

**Table 26:** Optimal solution, risk and return for the GRU model for λ=0.3 - Case study I.

	Tracking model	M-V without bootstrapping	M-V with real return data
Akhaber	**0.267**	0.000	0.000
Asia	**0.596**	**1.000**	0.000
Foulad	**0.010**	0.000	0.000
Hkshti	**0.010**	0.000	0.000
Mafakher	**0.009**	0.000	0.000
Nori	**0.011**	0.000	0.000
Satran	**0.025**	0.000	**1.000**
Shasta	**0.007**	0.000	0.000
Tejarat	**0.065**	0.000	0.000
Risk	1.5e-04	2.7e-04	9.7e-04
Return	5.5e-03	6.6e-03	5.6e-03

**Table 27:** Optimal solution, risk and return for the GRU model for λ=0.4 - Case study I.

	Tracking model	M-V without bootstrapping	M-V with real return data
Akhaber	**0.218**	0.000	0.000
Asia	**0.561**	**1.000**	0.000
Foulad	**0.021**	0.000	0.000
Hkshti	**0.020**	0.000	0.000
Mafakher	**0.017**	0.000	0.000
Nori	**0.021**	0.000	0.000
Satran	**0.048**	0.000	**1.000**
Shasta	**0.011**	0.000	0.000
Tejarat	**0.083**	0.000	0.000
Risk	1.3e-04	2.7-04	9.7e-04
Return	5.3e-03	6.6e-03	5.6e-03

**Table 28:** Optimal solution, risk and return for the GRU model for λ=0.5 - Case study I.

	Tracking model	M-V without bootstrapping	M-V with real return data
Akhaber	**0.217**	0.000	**0.138**
Asia	**0.568**	**1.000**	0.000
Foulad	**0.019**	0.000	0.000
Hkshti	**0.018**	0.000	0.000
Mafakher	**0.016**	0.000	0.000
Nori	**0.019**	0.000	0.000
Satran	**0.043**	0.000	**0.862**
Shasta	**0.013**	0.000	0.000
Tejarat	**0.087**	0.000	0.000
Risk	1.3e-04	2.7e-04	7.3e-04
Return	5.4e-03	6.6e-03	5.5e-03

**Table 29:** Optimal solution, risk and return for the GRU model for λ=0.6 - Case study I.

	Tracking model	M-V without bootstrapping	M-V with real return data
Akhaber	**0.225**	0.000	**0.265**
Asia	**0.495**	**1.000**	0.000
Foulad	**0.025**	0.000	0.000
Hkshti	**0.026**	0.000	0.000
Mafakher	**0.023**	0.000	0.000
Nori	**0.028**	0.000	0.000
Satran	**0.059**	0.000	**0.734**
Shasta	**0.0019**	0.000	0.000
Tejarat	**0.099**	0.000	0.000
Risk	1.1e-04	2.7e-04	5.8e-04
Return	5.2e-03	6.6e-03	5.4e-03

**Table 30:** Optimal solution, risk and return for the GRU model for λ=0.7 - Case study I.

	Tracking model	M-V without bootstrapping	M-V with real return data
Akhaber	**0.263**	0.000	**0.356**
Asia	**0.484**	**1.000**	0.000
Foulad	**0.020**	0.000	0.000
Hkshti	**0.020**	0.000	0.000
Mafakher	**0.017**	0.000	0.000
Nori	**0.022**	0.000	0.000
Satran	**0.051**	0.000	**0.644**
Shasta	**0.015**	0.000	0.000
Tejarat	**0.108**	0.000	0.000
Risk	1.1e-05	2.7e-04	5.0e-04
Return	5.2e-03	6.6e-03	5.3e-03

**Table 31:** Optimal solution, risk and return for the GRU model for λ=0.8 - Case study I.

	Tracking model	M-V without bootstrapping	M-V with real return data
Akhaber	**0.168**	**0.935**	**0.424**
Asia	**0.286**	**0.065**	0.000
Foulad	**0.063**	0.000	0.000
Hkshti	**0.067**	0.000	0.000
Mafakher	**0.060**	0.000	0.000
Nori	**0.073**	0.000	0.000
Satran	**0.108**	0.000	**0.576**
Shasta	**0.048**	0.000	0.000
Tejarat	**0.125**	0.000	0.000
Risk	3.4e-05	2.4e-04	4.7e-04
Return	4.3e-03	6.6e-03	5.3e-03

**Table 32:** Optimal solution, risk and return for the GRU model for λ=0.9 - Case study I.

	Tracking model	M-V without bootstrapping	M-V with real return data
Akhaber	**0.111**	0.000	**0.445**
Asia	**0.110**	**0.618**	0.000
Foulad	**0.117**	**0.382**	0.000
Hkshti	**0.111**	0.000	0.000
Mafakher	**0.111**	0.000	0.000
Nori	**0.117**	0.000	0.000
Satran	**0.100**	**0.018**	**0.498**
Shasta	**0.110**	0.000	0.000
Tejarat	**0.111**	0.000	0.000
Risk	3.4e-05	1.4e-05	4.3e-04
Return	3.5e-03	6.3e-03	5.1e-03

**Table 33:** Optimal solution, risk and return for the CNN model for λ=0.1 - Case study II.

	Tracking model	M-V without bootstrapping	M-V with real return data
Khodro	**0.0667**	0.0000	0.0000
Foulad	**0.0667**	0.0000	0.0000
Shepna	**0.0667**	0.0000	**1.0000**
Kgl	**0.0667**	0.0000	0.0000
Sefars	**0.0667**	0.0000	0.0000
Drouz	**0.0667**	0.0000	0.0000
Bswitch	**0.0667**	0.0000	0.0000
Kkhak	**0.0667**	0.0000	0.0000
Hkshti	**0.0667**	**1.0000**	0.0000
Ghshasfa	**0.0667**	0.0000	0.0000
Sakht	**0.0667**	0.0000	0.0000
Ghsabet	**0.0667**	0.0000	0.0000
Sesoufi	**0.0667**	0.0000	0.0000
Khsapa	**0.0667**	0.0000	0.0000
Fameli	**0.0667**	0.0000	0.0000
Risk	8.15e-06	8.55e-05	5.75e-04
Return	1.63e-03	5.86e-03	2.20e-03

**Table 34:** Optimal solution, risk and return for the CNN model for λ=0.2 - Case study II.

	Tracking model	M-V without bootstrapping	M-V with real return data
Khodro	**0.0667**	0.0000	0.0000
Foulad	**0.0667**	0.0000	0.0000
Shepna	**0.0667**	0.0000	**1.0000**
Kgl	**0.0667**	0.0000	0.0000
Sefars	**0.0667**	0.0000	0.0000
Drouz	**0.0667**	0.0000	0.0000
Bswitch	**0.0667**	0.0000	0.0000
Kkhak	**0.0667**	0.0000	0.0000
Hkshti	**0.0667**	**1.0000**	0.0000
Ghshasfa	**0.0667**	0.0000	0.0000
Sakht	**0.0667**	0.0000	0.0000
Ghsabet	**0.0667**	0.0000	0.0000
Sesoufi	**0.0667**	0.0000	0.0000
Khsapa	**0.0667**	0.0000	0.0000
Fameli	**0.0667**	0.0000	0.0000
Risk	8.15e-06	8.55e-05	5.75e-04
Return	1.63e-03	5.86e-03	2.20e-03

**Table 35:** Optimal solution, risk and return for the CNN model for λ=0.3 - Case study II.

	Tracking model	M-V without bootstrapping	M-V with real return data
Khodro	**0.0667**	0.0000	**0.0003**
Foulad	**0.0667**	0.0000	0.0000
Shepna	**0.0667**	0.0000	**0.9997**
Kgl	**0.0667**	0.0000	0.0000
Sefars	**0.0667**	0.0000	0.0000
Drouz	**0.0667**	0.0000	0.0000
Bswitch	**0.0667**	0.0000	0.0000
Kkhak	**0.0667**	0.0000	0.0000
Hkshti	**0.0667**	**1.0000**	0.0000
Ghshasfa	**0.0667**	0.0000	0.0000
Sakht	**0.0667**	0.0000	0.0000
Ghsabet	**0.0667**	0.0000	0.0000
Sesoufi	**0.0667**	0.0000	0.0000
Khsapa	**0.0667**	0.0000	0.0000
Fameli	**0.0667**	0.0000	0.0000
Risk	8.15e-06	8.55e-05	5.74e-04
Return	1.63e-03	5.86e-03	2.20e-03

**Table 36:** Optimal solution, risk and return for the CNN model for λ=0.4 - Case study II.

	Tracking model	M-V without bootstrapping	M-V with real return data
Khodro	**0.0667**	0.0000	**0.1667**
Foulad	**0.0667**	0.0000	0.0000
Shepna	**0.0667**	0.0000	**0.8332**
Kgl	**0.0667**	0.0000	0.0000
Sefars	**0.0667**	0.0000	0.0000
Drouz	**0.0667**	0.0000	0.0000
Bswitch	**0.0667**	0.0000	0.0000
Kkhak	**0.0667**	0.0000	0.0000
Hkshti	**0.0667**	**1.0000**	0.0000
Ghshasfa	**0.0667**	0.0000	0.0000
Sakht	**0.0667**	0.0000	0.0000
Ghsabet	**0.0667**	0.0000	0.0000
Sesoufi	**0.0667**	0.0000	0.0000
Khsapa	**0.0667**	0.0000	0.0000
Fameli	**0.0667**	0.0000	0.0000
Risk	8.15e-06	8.55e-05	4.05e-04
Return	1.63e-03	5.86e-03	2.10e-03

**Table 37:** Optimal solution, risk and return for the CNN model for λ=0.5 - Case study II.

	Tracking model	M-V without bootstrapping	M-V with real return data
Khodro	**0.0667**	0.0000	**0.2664**
Foulad	**0.0667**	0.0000	0.0000
Shepna	**0.0667**	0.0000	**0.6711**
Kgl	**0.0667**	0.0000	0.0000
Sefars	**0.0667**	0.0000	0.0000
Drouz	**0.0667**	0.0000	0.0000
Bswitch	**0.0667**	0.0000	0.0000
Kkhak	**0.0667**	0.0000	0.0000
Hkshti	**0.0667**	**1.0000**	0.0000
Ghshasfa	**0.0667**	0.0000	0.0000
Sakht	**0.0667**	0.0000	0.0000
Ghsabet	**0.0667**	0.0000	0.0000
Sesoufi	**0.0667**	0.0000	**0.0625**
Khsapa	**0.0667**	0.0000	0.0000
Fameli	**0.0667**	0.0000	0.0000
Risk	8.15e-06	8.55e-05	3.00e-04
Return	1.63e-03	5.86e-03	2.02e-03

**Table 38:** Optimal solution, risk and return for the CNN model for λ=0.6 - Case study II.

	Tracking model	M-V without bootstrapping	M-V with real return data
Khodro	**0.0667**	0.0000	**0.3160**
Foulad	**0.0667**	0.0000	0.0000
Shepna	**0.0667**	0.0000	**0.5378**
Kgl	**0.0667**	0.0000	0.0000
Sefars	**0.0667**	0.0000	0.0000
Drouz	**0.0667**	0.0000	0.0000
Bswitch	**0.0667**	0.0000	0.0000
Kkhak	**0.0667**	0.0000	0.0000
Hkshti	**0.0667**	**1.0000**	0.0000
Ghshasfa	**0.0667**	0.0000	0.0000
Sakht	**0.0667**	0.0000	0.0000
Ghsabet	**0.0667**	0.0000	0.0000
Sesoufi	**0.0667**	0.0000	**0.1461**
Khsapa	**0.0667**	0.0000	0.0000
Fameli	**0.0667**	0.0000	0.0000
Risk	8.15e-06	8.55e-05	2.44e-04
Return	1.63e-03	5.86e-03	1.95e-03

**Table 39:** Optimal solution, risk and return for the CNN model for λ=0.7 - Case study II.

	Tracking model	M-V without bootstrapping	M-V with real return data
Khodro	**0.0667**	0.0000	**0.3467**
Foulad	**0.0667**	0.0000	0.0000
Shepna	**0.0667**	0.0000	**0.4386**
Kgl	**0.0667**	0.0000	0.0000
Sefars	**0.0667**	0.0000	0.0000
Drouz	**0.0667**	0.0000	0.0000
Bswitch	**0.0667**	0.0000	0.0000
Kkhak	**0.0667**	0.0000	0.0000
Hkshti	**0.0667**	**1.0000**	0.0000
Ghshasfa	**0.0667**	0.0000	**0.0133**
Sakht	**0.0667**	0.0000	0.0000
Ghsabet	**0.0667**	0.0000	0.0000
Sesoufi	**0.0667**	0.0000	**0.2013**
Khsapa	**0.0667**	0.0000	0.0000
Fameli	**0.0667**	0.0000	0.0000
Risk	8.15e-06	8.55e-05	2.13e-04
Return	1.63e-03	5.86e-03	1.89e-03

**Table 40:** Optimal solution, risk and return for the CNN model for λ=0.8 - Case study II.

	Tracking model	M-V without bootstrapping	M-V with real return data
Khodro	**0.0667**	0.0000	**0.3076**
Foulad	**0.0667**	0.0000	0.0000
Shepna	**0.0667**	0.0000	**0.3135**
Kgl	**0.0667**	0.0000	0.0000
Sefars	**0.0667**	0.0000	0.0000
Drouz	**0.0667**	0.0000	**0.0006**
Bswitch	**0.0667**	0.0000	0.0000
Kkhak	**0.0667**	0.0000	0.0000
Hkshti	**0.0667**	**1.0000**	0.0000
Ghshasfa	**0.0667**	0.0000	**0.1958**
Sakht	**0.0667**	0.0000	0.0000
Ghsabet	**0.0667**	0.0000	0.0000
Sesoufi	**0.0667**	0.0000	**0.1824**
Khsapa	**0.0667**	0.0000	0.0000
Fameli	**0.0667**	0.0000	0.0000
Risk	8.15e-06	8.55e-05	1.56e-04
Return	1.63e-03	5.86e-03	1.72e-03

**Table 41:** Optimal solution, risk and return for the CNN model for λ=0.9 - Case study II.

	Tracking model	M-V without bootstrapping	M-V with real return data
Khodro	**0.0667**	0.0000	**0.2198**
Foulad	**0.0667**	0.0000	0.0000
Shepna	**0.0667**	0.0000	**0.2074**
Kgl	**0.0667**	0.0000	0.0000
Sefars	**0.0667**	0.0000	0.0000
Drouz	**0.0667**	0.0000	**0.2266**
Bswitch	**0.0667**	0.0000	0.0000
Kkhak	**0.0667**	0.0000	0.0000
Hkshti	**0.0667**	**0.9957**	0.0000
Ghshasfa	**0.0667**	0.0000	**0.2070**
Sakht	**0.0667**	0.0000	0.0000
Ghsabet	**0.0667**	0.0000	0.0000
Sesoufi	**0.0667**	0.0000	**0.1390**
Khsapa	**0.0667**	0.0000	0.0000
Fameli	**0.0667**	0.0000	0.0000
Risk	8.15e-06	8.48e-05	7.65e-05
Return	1.63e-03	5.86e-03	1.26e-03

**Table 42:** Optimal solution, risk and return for the GRU model for λ=0.1 - Case study II.

	Tracking model	M-V without bootstrapping	M-V with real return data
Khodro	**0.0103**	0.0000	0.0000
Foulad	**0.0109**	0.0000	0.0000
Shepna	**0.0094**	0.0000	**1.0000**
Kgl	**0.0053**	0.0000	0.0000
Sefars	**0.0126**	0.0000	0.0000
Drouz	**0.0067**	0.0000	0.0000
Bswitch	**0.0075**	0.0000	0.0000
Kkhak	**0.0269**	0.0000	0.0000
Hkshti	**0.0951**	**1.0000**	0.0000
Ghshasfa	**0.0084**	0.0000	0.0000
Sakht	**0.0134**	0.0000	0.0000
Ghsabet	**0.0080**	0.0000	0.0000
Sesoufi	**0.7670**	0.0000	0.0000
Khsapa	**0.0083**	0.0000	0.0000
Fameli	**0.0102**	0.0000	0.0000
Risk	1.22e-04	3.43e-04	5.75e-04
Return	4.18e-03	6.30e-03	2.20e-03

**Table 43:** Optimal solution, risk and return for the GRU model for λ=0.2 - Case study II.

	Tracking model	M-V without bootstrapping	M-V with real return data
Khodro	**0.0103**	0.0000	0.0000
Foulad	**0.0112**	0.0000	0.0000
Shepna	**0.0096**	0.0000	**1.0000**
Kgl	**0.0053**	0.0000	0.0000
Sefars	**0.0130**	0.0000	0.0000
Drouz	**0.0071**	0.0000	0.0000
Bswitch	**0.0076**	0.0000	0.0000
Kkhak	**0.0238**	0.0000	0.0000
Hkshti	**0.1501**	**1.0000**	0.0000
Ghshasfa	**0.0086**	0.0000	0.0000
Sakht	**0.0138**	0.0000	0.0000
Ghsabet	**0.0083**	0.0000	0.0000
Sesoufi	**0.7123**	0.0000	0.0000
Khsapa	**0.0086**	0.0000	0.0000
Fameli	**0.0104**	0.0000	0.0000
Risk	1.11e-04	3.43e-04	5.75e-04
Return	4.11e-03	6.30e-03	2.20e-03

**Table 44:** Optimal solution, risk and return for the GRU model for λ=0.3 - Case study II.

	Tracking model	M-V without bootstrapping	M-V with real return data
Khodro	**0.0114**	0.0000	**0.0003**
Foulad	**0.0124**	0.0000	0.0000
Shepna	**0.0107**	0.0000	**0.9997**
Kgl	**0.0059**	0.0000	0.0000
Sefars	**0.0142**	0.0000	0.0000
Drouz	**0.0078**	0.0000	0.0000
Bswitch	**0.0084**	0.0000	0.0000
Kkhak	**0.0287**	0.0000	0.0000
Hkshti	**0.1513**	**1.0000**	0.0000
Ghshasfa	**0.0095**	0.0000	0.0000
Sakht	**0.0151**	0.0000	0.0000
Ghsabet	**0.0091**	0.0000	0.0000
Sesoufi	**0.6943**	0.0000	0.0000
Khsapa	**0.0095**	0.0000	0.0000
Fameli	**0.0115**	0.0000	0.0000
Risk	1.06e-04	3.43e-04	5.74e-04
Return	4.05e-03	6.30e-03	2.20e-03

**Table 45:** Optimal solution, risk and return for the GRU model for λ=0.4 - Case study II.

	Tracking model	M-V without bootstrapping	M-V with real return data
Khodro	**0.0136**	0.0000	**0.1667**
Foulad	**0.0151**	0.0000	0.0000
Shepna	**0.0131**	0.0000	**0.8332**
Kgl	**0.0071**	0.0000	0.0000
Sefars	**0.0178**	0.0000	0.0000
Drouz	**0.0097**	0.0000	0.0000
Bswitch	**0.0101**	0.0000	0.0000
Kkhak	**0.0345**	0.0000	0.0000
Hkshti	**0.1543**	**1.0000**	0.0000
Ghshasfa	**0.0117**	0.0000	0.0000
Sakht	**0.0185**	0.0000	0.0000
Ghsabet	**0.0112**	0.0000	0.0000
Sesoufi	**0.6573**	0.0000	0.0000
Khsapa	**0.0120**	0.0000	0.0000
Fameli	**0.0141**	0.0000	0.0000
Risk	9.70e-05	3.43e-04	4.05e-04
Return	3.93e-03	6.30e-03	2.10e-03

**Table 46:** Optimal solution, risk and return for the GRU model for λ=0.5 - Case study II.

	Tracking model	M-V without bootstrapping	M-V with real return data
Khodro	**0.0176**	0.0000	**0.2664**
Foulad	**0.0194**	0.0000	0.0000
Shepna	**0.0170**	0.0000	**0.6711**
Kgl	**0.0093**	0.0000	0.0000
Sefars	**0.0232**	0.0000	0.0000
Drouz	**0.0126**	0.0000	0.0000
Bswitch	**0.0132**	0.0000	0.0000
Kkhak	**0.0494**	0.0000	0.0000
Hkshti	**0.1813**	**1.0000**	0.0000
Ghshasfa	**0.0152**	0.0000	0.0000
Sakht	**0.0237**	0.0000	0.0000
Ghsabet	**0.0146**	0.0000	0.0000
Sesoufi	**0.5694**	0.0000	0.0000
Khsapa	**0.0156**	0.0000	0.0000
Fameli	**0.0184**	0.0000	0.0000
Risk	7.94e-05	3.43e-04	3.00e-04
Return	3.69e-03	6.30e-03	2.02e-03

**Table 47:** Optimal solution, risk and return for the GRU model for λ=0.6 - Case study II.

	Tracking model	M-V without bootstrapping	M-V with real return data
Khodro	**0.0667**	0.0000	**0.3160**
Foulad	**0.0667**	0.0000	0.0000
Shepna	**0.0667**	0.0000	**0.5378**
Kgl	**0.0667**	0.0000	0.0000
Sefars	**0.0667**	0.0000	0.0000
Drouz	**0.0667**	0.0000	0.0000
Bswitch	**0.0667**	0.0000	0.0000
Kkhak	**0.0667**	0.0000	0.0000
Hkshti	**0.0667**	**1.0000**	0.0000
Ghshasfa	**0.0667**	0.0000	0.0000
Sakht	**0.0667**	0.0000	0.0000
Ghsabet	**0.0667**	0.0000	0.0000
Sesoufi	**0.0667**	0.0000	**0.1461**
Khsapa	**0.0667**	0.0000	0.0000
Fameli	**0.0667**	0.0000	0.0000
Risk	1.88e-05	3.43e-04	2.44e-04
Return	1.09e-03	6.30e-03	1.95e-03

**Table 48:** Optimal solution, risk and return for the GRU model for λ=0.7 - Case study II.

	Tracking model	M-V without bootstrapping	M-V with real return data
Khodro	**0.0667**	0.0000	**0.3467**
Foulad	**0.0667**	0.0000	0.0000
Shepna	**0.0667**	0.0000	**0.4386**
Kgl	**0.0667**	0.0000	0.0000
Sefars	**0.0667**	0.0000	0.0000
Drouz	**0.0667**	0.0000	0.0000
Bswitch	**0.0667**	0.0000	0.0000
Kkhak	**0.0667**	0.0000	0.0000
Hkshti	**0.0667**	**0.9997**	0.0000
Ghshasfa	**0.0667**	0.0000	**0.0133**
Sakht	**0.0667**	0.0000	0.0000
Ghsabet	**0.0667**	0.0000	0.0000
Sesoufi	**0.0667**	0.0000	**0.2013**
Khsapa	**0.0667**	0.0000	0.0000
Fameli	**0.0667**	0.0000	0.0000
Risk	1.88e-05	3.43e-04	2.13e-04
Return	1.09e-03	6.30e-03	1.89e-03

**Table 49:** Optimal solution, risk and return for the GRU model for λ=0.8 - Case study II.

	Tracking model	M-V without bootstrapping	M-V with real return data
Khodro	**0.0667**	0.0000	**0.3076**
Foulad	**0.0667**	0.0000	0.0000
Shepna	**0.0667**	0.0000	**0.3135**
Kgl	**0.0667**	0.0000	0.0000
Sefars	**0.0667**	0.0000	0.0000
Drouz	**0.0667**	0.0000	**0.0006**
Bswitch	**0.0667**	0.0000	0.0000
Kkhak	**0.0667**	0.0000	0.0000
Hkshti	**0.0667**	**0.7687**	0.0000
Ghshasfa	**0.0667**	0.0000	**0.1958**
Sakht	**0.0667**	0.0000	0.0000
Ghsabet	**0.0667**	0.0000	0.0000
Sesoufi	**0.0667**	**0.2313**	**0.1824**
Khsapa	**0.0667**	0.0000	0.0000
Fameli	**0.0667**	0.0000	0.0000
Risk	1.88e-05	2.06e-04	1.56e-04
Return	1.09e-03	5.86e-03	1.72e-03

**Table 50:** Optimal solution, risk and return for the GRU model for λ=0.9 - Case study II.

	Tracking model	M-V without bootstrapping	M-V with real return data
Khodro	**0.0667**	0.0000	**0.2198**
Foulad	**0.0667**	0.0000	0.0000
Shepna	**0.0667**	0.0000	**0.2074**
Kgl	**0.0667**	0.0000	0.0000
Sefars	**0.0667**	0.0000	0.0000
Drouz	**0.0667**	0.0000	**0.2266**
Bswitch	**0.0667**	0.0000	0.0000
Kkhak	**0.0667**	0.0000	0.0000
Hkshti	**0.0667**	**0.5146**	0.0000
Ghshasfa	**0.0667**	0.0000	**0.2070**
Sakht	**0.0667**	0.0000	0.0000
Ghsabet	**0.0667**	0.0000	0.0000
Sesoufi	**0.0667**	**0.4854**	**0.1390**
Khsapa	**0.0667**	0.0000	0.0000
Fameli	**0.0667**	0.0000	0.0000
Risk	1.88e-05	1.19e-04	7.65e-05
Return	1.09e-03	5.38e-03	1.26e-03

## Supporting information

S1 Data and Codes (RAR)
